# Global, regional, and national burden of ischemic heart disease attributable to secondhand smoke from 1990 to 2019

**DOI:** 10.18332/tid/189771

**Published:** 2024-07-04

**Authors:** Xinyue Yang, Zhiqiang Zhang, Jiayi Sun, Wenjuan Zhang

**Affiliations:** 1 Department of Cardiovascular Medicine, Tianjin Medical University General Hospital, Tianjin, China

**Keywords:** global burden of disease, secondhand smoke, ischemic heart disease, disability-adjusted life-years

## Abstract

**INTRODUCTION:**

Assessing the burden of ischemic heart disease (IHD) attributable to secondhand smoke (SHS) exposure is crucial for informing evidence-based healthcare practices, prevention strategies, and resource allocation planning.

**METHODS:**

The burden of IHD attributable to SHS from 1990 to 2019 was assessed using the comparative risk assessment method as part of the Global Burden of Disease (GBD) study 2019.

**RESULTS:**

Globally, the absolute number of deaths and disability-adjusted life-years (DALYs) from IHD due to SHS increased substantially from 270.0 thousand and 6971.3 thousand in 1990 to 397.4 thousand and 9566.1 thousand in 2019. The corresponding age-standardized mortality rates (ASMR) and age-standardized DALYs rates (ASDR) were both in a decreasing trend with estimate of the annual percentage change (EAPC) of -1.38 (-1.42 – -1.34) and -1.43 (-1.47 – -1.38). Central Asia has the highest ASMR (16 per 100000, 95% uncertainty interval, UI: 12.8–19.4), and Oceania has the highest ASDR (323.2 per 100000, 95% UI: 228.9–443.1 per 100000) in 2019. All sociodemographic index (SDI) category regions showed a decreasing trend in ASMR and ASDR, with the decrease being more obvious in high and high-middle SDI regions. Our analysis identified an escalating trend concerning ASMR and ASDR in Oceania from 1990 to 2019. In 2019, the most significant number of deaths and DALYs occurred in the age group of 80–84 years (5.4 thousand, 95% UI: 3.7–7.3 in thousands) and the age group of 55–59 years (1140.8 thousand, 95% UI: 876.1–1435 in thousands).

**CONCLUSIONS:**

Our study reveals an absolute global increase in deaths and DALYs from IHD due to SHS from 1990 to 2019. Despite a declining trend in ASMR and ASDR, regional disparities persist. The elderly and middle-aged populations bore the most significant burden. These findings highlight the ongoing global health impact of SHS on IHD and emphasize the need for targeted interventions in regions with rising trends and vulnerable age groups.

## INTRODUCTION

Ischemic heart disease (IHD), a leading global health burden, has long been a significant contributor to mortality and disability^1^. The Global Burden of Disease (GBD) Study reports that IHD is responsible for approximately 9.13 million deaths and 180 million disability-adjusted life years (DALYs) in 2019^2^, which not only inflicts considerable suffering and economic burden but also poses a formidable challenge to public health systems worldwide^2^.

In recent years, with deepening awareness of the harms of smoking, public attention to the connection between active smoking and cardiovascular diseases has increased^3^. However, the harm caused by secondhand smoke (SHS), compared to direct smoking, is often underestimated^3^. SHS, the smoke inhaled by non-smokers from the burning of tobacco products, includes smoke exhaled by smokers and smoke directly released into the air from burning tobacco products^4^. Numerous studies have demonstrated that individuals exposed to SHS environments over a long term have a significantly increased risk of cardiovascular diseases^3^. The toxic substances contained in SHS can directly damage the cardiovascular system, augmenting the risk of heart disease^3^.

Previous studies have explored the association between SHS and IHD^5,6^, yet limited research has evaluated the spatial and temporal trends of IHD burden attributable to SHS globally. This study comprehensively assesses the impact of SHS exposure on the IHD burden by analyzing data from 204 countries and regions from 1990 to 2019, aiming to provide a scientific basis for formulating effective public health policies and interventions to mitigate the deleterious effects of SHS on cardiovascular health.

## METHODS

### Data sources

This is a secondary dataset analysis from the GBD 2019 database (http://ghdx.healthdata.org/gbd-results-tool ), encompassing statistics from 204 countries and regions on 369 diseases and 87 risk factors from 1990 to 2019^7^. The GBD provides detailed data on the burden of IHD attributable to SHS, categorized by gender, age, region, and country, and further divided into five sociodemographic index (SDI) categories: high, high-middle, middle, low-middle, and low^8^. The SDI is a composite measure of a location’s sociodemographic development, calculated as the geometric mean of normalized values for income per capita, education level, and total fertility rate. The GBD Study categorizes the world into 21 geographical regions based on epidemiological similarities and geographical proximity, such as East Asia, Western Europe, and Eastern Europe^9^. Further details of the data sources used in this analysis are available on the GBD 2019 Sources Tool website.

### Definition of secondhand smoke exposure

The GBD 2019 Risk Factors Collaborators conducted a standardized and comprehensive evaluation of the exposure levels to risk factors, the relative risks, and the associated disease burden^10^. SHS exposure was defined as current exposure to secondhand tobacco smoke at home, at work, or in other public places. Household composition was used as a proxy for non-occupational SHS exposure, assuming that all persons living with a daily smoker are exposed to tobacco smoke. Surveys were used to gauge the percentage of people who were exposed to secondhand smoke in the workplace. For this study, individuals who did not smoke daily, former smokers, and those who smoked occasionally were classified as non-smokers and assumed to have been exposed to secondhand smoke. Assessment is conducted for both children and grown-ups.

Household composition data, including the ages and genders of all residents, were used to determine the percentage of non-smokers living with at least one smoker. Data sources utilized various major survey series that incorporated a household composition module, such as the Demographic and Health Surveys, the Multiple Indicator Cluster Surveys, and the Living Standards Measurement Surveys, in addition to national and subnational censuses, including those included in the IPUMS project and identified through the Global Health Data Exchange (GHDx) catalog. Surveys were utilized to determine the percentage of people exposed to secondhand smoke in the workplace based on age and gender through self-reported information. Data were gathered from various sources such as the Global Adult Tobacco Surveys, Eurobarometer Surveys, and WHO STEPS Surveys. These sources were identified using the GHDx.

GBD 2019 assessed risk factor exposures using representative survey and surveillance data and geospatial Gaussian process regression models that leveraged temporal and geographical correlations for enhanced accuracy. The relative risk obtained from meta-analyses was applied to the population exposure distributions to estimate the proportion of IHD cases attributable to secondhand smoke. Additionally, advanced statistical techniques, including geospatial Gaussian process regression models and Bayesian meta-regression adjusted for potential confounders and comorbidities were used to ensure robust and accurate estimates. These methods are consistent with those described in previous studies^1^.

### Statistical analysis

Using data from the GBD 2019, we estimated the number of deaths and DALYs for the burden of IHD attributable to SHS exposure annually from 1990 to 2019, allowing for a comprehensive trend analysis over the 30 years. The GBD study has already outlined the procedures for determining mortality rates (number of deaths per 100000 people) and DALYs (total years lost due to early death and years lived with disability) with their 95% uncertainty intervals (UI)^10^. The 95% UI is estimated through uncertainty analysis involving different models and parameter settings. This typically includes several steps, such as using Bayesian statistical models for correction and adjustment, generating risk estimates under various scenarios, and combining these estimates to derive the final 95% UI. Comparability is guaranteed by using age-standardized rates (ASR) and their 95% UI to account for variations in age distributions among populations and over time^11^. Therefore, this study employs age-standardized mortality rates (ASMR) and age-standardized DALY rates (ASDR) to illustrate the variations in the IHD burden attributable to SHS exposure across different regions.

We analyze the trends in ASR from 1990 to 2019 by using the estimate of the annual percentage change (EAPC). The EAPC is derived from the logarithmic, linear regression equation of ASR, ln (ASR) = α + βX + ε, where β=regression coefficient, X=each calendar year within the period from 1990 to 2019, and the EAPC is calculated as 100 × [exp(β) – 1]^12^. If the lower limit of the 95% confidence interval (CI) for the EAPC is greater than 0, the ASR is considered to be on an upward trend; if the upper limit of the 95% CI is less than 0, the ASR is considered to be on a downward trend; if the 95% CI includes both positive and negative numbers, the ASR is considered stable. All statistical analyses were performed using R software (version 4.3.1).

## RESULTS

### Ischemic heart disease deaths and ASMR associated with secondhand smoke

**Figure 1:**
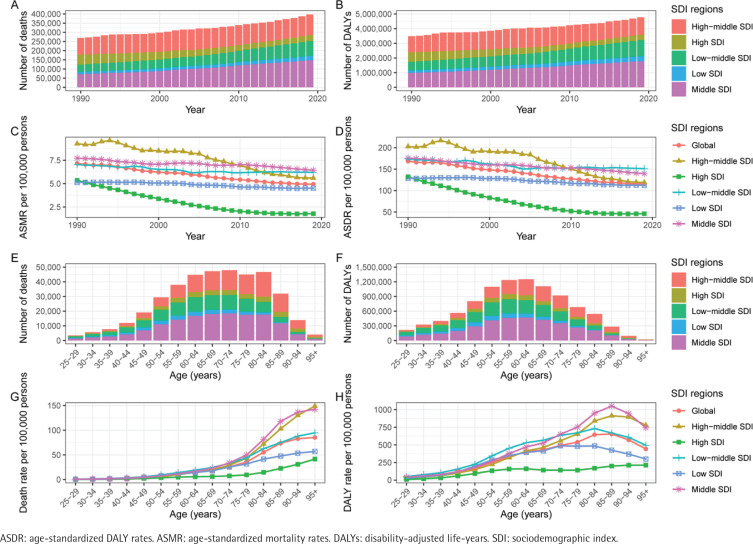
The ischemic heart disease burden attributable to secondhand smoke by SDI region. The global (A) deaths, (B) DALYs, (C) ASMR and (D) ASDR of ischemic heart disease attributable to secondhand smoke for all ages from 1990 to 2019. The global (E) deaths, (F) DALYs, (G) mortality rate and (H) DALYs rate of ischemic heart disease attributable to secondhand smoke by age in 2019

Globally, the total number of IHD-related deaths due to SHS increased substantially from 1990 (270.0 thousand, 95% UI: 219.6–323.1 in thousands) to 2019 (397.4 thousand, 95% UI: 319.9–477.6 in thousands) (Table 1, Figure 1A). Although the total number of deaths has significantly increased, the global ASMR has shown a downward trend, dropping from 7.2 per 100000 population in 1990 (95% UI: 5.9–8.5) to 4.9 per 100000 population in 2019 (95% UI: 4.0–4.9), with an EAPC of -1.38 (95% CI: -1.42 – -1.34) (Table 1, Figure 1C).

**Table 1:** The global ischemic heart disease burden attributable to secondhand smoke in 1990 and 2019, and the temporal trends from 1990 to 2019

Characteristics	1990				2019				EAPC (1990–2019)	
	Death cases n×10 ^3^ (95% UI)	ASMR per 10 ^5^ n (95% UI)	DALYs n×10 ^3^ (95% UI)	ASDR per 10 ^5^ n (95% UI)	Death cases n×10 ^3^ (95% UI)	ASMR per 10 ^5^ n (95% UI)	DALYs, n×10 ^3^ (95% UI)	ASDR per 10 ^5^ n (95% UI)	ASMR n (95% CI)	ASDR n (95% CI)
**Global**	270 219.6–323.1	7.2 5.9–8.5	6971.3 5667–8351.5	168.5 136.7–201.8	397.4 319.9–477.6	4.9 4–5.9	9566.1 7720.3–11527.1	115.5 93.3–139	-1.38 -1.42 – -1.34	-1.43 -1.47 – -1.38
**Sex**										
Male	131.2 106.5–157.8	7.6 6.2–9.1	3610.2 2915.8–4341.6	182.3 147.9–218.7	193.6 153.6–235.4	5.2 4.1–6.4	4970.7 3956.9–6048.4	124.8 99.6–151.7	-1.36 -1.4 – -1.32	-1.38 -1.42 – -1.34
Female	138.7 112.7–165.3	6.7 5.5–8	3361.2 2734.6–4010.2	155 126.2–184.8	203.8 164.3–248.1	4.7 3.8–5.7	4595.4 3741.2–5541.4)	106.3 86.3–128.1	-1.4 -1.45 – -1.35	-1.48 -1.55 – -1.41
**SDI**										
High	54.6 44.9–64.6	5.3 4.4–6.3	1307.9 1086.9–1556.4	132.1 109.2–156.2	32.4 26.5–38.4	1.8 1.5–2.1	727.1 596.8–862.1	46 37.7–54.9	-4.17 -4.41 – -3.93	-3.97 -4.21 – -3.73
High-middle	91.7 **75–109.2**	9.3 7.6–11	2186.3 1785–2607.6	202.6 166.2–241.4	112.2 90.9–135.8	5.6 4.5–6.8	2388.7 1944.8–2874.3	119 96.9–142.8	-1.98 -2.17 – -1.79	-2.16 -2.4 – -1.92
Middle	71.8 **58–86.4**	7.7 6.2–9.3	1955.5 1587–2335.8	175.9 142.7–210.7	147.9 117.8–178.5	6.4 5.1–7.8)	3558.5 2858.7–4299	139.7 112.4–167.6	-0.51 -0.57 – -0.45	-0.72 -0.76 – -0.68
Low-middle	40 31.7–48.4	7 5.6–8.5	1172.3 925.8–1420.4	173.9 137.7–210.2	81.7 64.9–99.4	6.2 4.9–7.5	2212.9 1764.8–2699.9	151.1 120.2–183.8	-0.47 -0.55 – -0.38	-0.5 -0.6 – -0.41
Low	11.7 9–14.6	5.1 4–6.4	345.5 268.5–433.3	128.5 100.5–160.8	23 17.9–28.3	4.5 3.5–5.6	673.3 518–832.4	112.5 87.3–137.8	-0.57 -0.63 – -0.52	-0.6 -0.67 – -0.53
**Region**										
Andean Latin America	0.5 0.4–0.6	2.5 2–3.2	13.8 10.5–17.4	60.6 46.2–76.8	0.6 0.4–0.8	1.1 0.8–1.4	14.8 10.3–19.7	25.4 17.7–33.7	-3.23 -3.56 – -2.9	-3.23 -3.54 – -2.92
Australasia	1.2 **1–1.5**	5.3 4.3–6.5	29.5 23.7–35.9	129.9 104.9–158.1	0.6 0.5–0.7	1.2 **1–1.5**	12.6 10.1–15.4	29.6 23.6–36.2	-5.47 -5.74 – -5.19	-5.36 -5.63 – -5.08
Caribbean	1.7 1.4-2	6.7 5.5–8.1	42.4 34.5–50.8	158.5 128.8–189.3	1.8 1.4–2.3	3.6 2.7–4.4	45 34.3–57.2	87.1 66.2–110.4	-2.47 -2.76 – -2.19	-2.3 -2.6 – -2.01
Central Asia	6.8 5.5–8	15.7 12.8–18.6	158.6 129.2–187.8	334.2 274.6–393.8	9.9 8–12	16 12.8–19.4	235.8 190.1–290.5	318.5 257.6–387.6	-0.28 -0.66–0.11	-0.6 -1.02 – -0.19
Central Europe	20.1 16.4–23.8	14.5 12–17.1	450.8 372.7–534.9	311.6 258.8–369.2	13.8 10.6–17.2	6.4 **5–7.8**	254.9 197.4–312.2	126.9 98.7–155	-3.26 -3.4 – -3.11	-3.57 -3.73 – -3.41
Central Latin America	3.6 2.8–4.5	4.6 3.6–5.7	93 72.6–113.4	103.4 81.1–126.6	6.1 4.5–7.8	2.6 1.9–3.4	142.6 103.9–184.7	58.9 42.9–76.5	-2.3 -2.51 – -2.09	-2.26 -2.47 – -2.04
Central Sub-Saharan Africa	0.5 0.4–0.7	2.4 1.7–3.2	16.2 11.7–21.8	61.2 44.7–81.7	1 0.7–1.4	1.9 1.3–2.6	29.8 20.1–42.7	46.6 31.8–66.1	-1 -1.07 – -0.94	-1.07 -1.14 – -1
East Asia	46 35.9–56.5	6.2 4.9–7.5	1219.1 955.3–1494.3	133.4 105–162.8	108.9 85.1–136.3	5.9 4.7–7.4	2268.8 1769.9–2846.9	112.7 88–140.6	0.35 0.12–0.57	-0.23 -0.39 – -0.07
Eastern Europe	30.3 24.3–36.6	11.5 9.3–13.8	674.3 543.1–814.5	245.2 199.5–292.9	33.2 26.8–40.4	9.6 7.8–11.7	711.5 572.6–866.4	215.4 173.7–261.9	-1.14 -1.7 – -0.58	-1.08 -1.74 – -0.41
Eastern Sub-Saharan Africa	1.5 1.2–2	2.1 1.6–2.7	45.3 33.6–58.5	53 39.8–67.6	2.8 2–3.8	1.8 1.3–2.4	83.3 59–112	43.4 30.4–57.8	-0.77 -0.81 – -0.73	-0.85 -0.89 – -0.8
High-income Asia Pacific	5.8 4.7–6.9	3 2.5–3.6	137.6 113.9–164.6	68.3 56.5–81.5	3.8 2.9–4.7	0.8 0.7–1	70.2 56.3–85.3	19.9 16.1–23.8	-4.44 -4.67 – -4.22	-4.14 -4.33 – -3.94
High-income North America	20.7 17.1–24.6	6.1 5.1–7.3	512.4 425.3–607.7	159.9 132.2–190.1	12.4 10.1–14.9	2.1 1.7–2.5	293.6 238.6–355.3	54.2 44.2–65.2	-4.35 -4.65 – -4.04	-4.25 -4.54 – -3.95
North Africa and Middle East	30.6 24.9–36.4	18.9 15.3–22.5	840.7 686.9–1009.2	447.3 365.7–532.9	47.4 37.3–58.2	11.5 9.1–14	1251.9 978.3–1543.6	264 206.6–324.1	-1.9 -1.99 – -1.81	-2.03 -2.12 – -1.94
Oceania	0.3 0.2–0.5	11.1 8.2–15.2	10.6 7.6–14.5	295.8 214.4–401.6	0.9 0.6–1.2	12.1 8.6–16.3	28.3 19.8–39.4	323.2 228.9–443.1	0.4 0.31–0.49	0.42 0.32–0.53
South Asia	41.8 32.3–51.2	7.8 6.1–9.5	1252.8 982.5–1545	192.2 149.5–235.4	93.3 73–115.7	6.8 5.3–8.4	2596.9 2019–3214.9	170.6 131.8–210.5	-0.6 -0.7 – -0.5	-0.51 -0.62 – -0.4
Southeast Asia	14.9 11.8–18.3	6.2 4.9–7.7	419.5 333.6–515.7	147.1 116.6–180.7	32 25.5–38.8	5.6 4.4–6.7	849.2 677.9–1039.7	130.2 103.8–158.2	-0.38 -0.46 – -0.31	-0.36 -0.44 – -0.28
Southern Latin America	3.7 3–4.5	8.5 6.9–10.2	80.8 64.8–96.7	176 141.6–210.4	2.6 2.1–3.1	3.1 2.5–3.8	53.9 43.3–65.3	66.3 53.2–80.4	-3.6 -3.81 – -3.38	-3.49 -3.68 – -3.31
Southern Sub-Saharan Africa	1.2 1–1.5	4.7 3.7–5.6	34.2 27.2–41.6	112.4 89.1–136.1	1.8 1.4–2.2	3.3 2.6–4.1	45 34.8–56.2	75.2 58.6–93.2	-1.17 -1.49 – -0.85	-1.38 -1.72 – -1.05
Tropical Latin America	7.6 6.2–9.1	8.6 7–10.2	214 175.2–256.2	211.7 172.9–253.8	7.1 5.7–8.7	2.9 2.4–3.6	187.5 150.1–228.8	74.8 60–91.4	-3.7 -3.88 – -3.51	-3.6 -3.76 – -3.43
Western Europe	28.4 23.3–33.8	5.1 4.2–6	660.3 544–784.6	125.6 103.6–149.7	12.9 10.4–15.5	1.5 1.2–1.7	264.4 216.4–314	35.4 28.9–42.2	-4.69 -4.88 – -4.5	-4.7 -4.89 – -4.52
Western Sub-Saharan Africa	2.5 1.8–3.3	3.1 2.2–4.1	65.4 47.8–89.8	69.7 51.3–94.2	4.6 3.5–6	2.7 2–3.4	125.9 93.5–165.7	60.1 45.4–77.9	-0.53 -0.62 – -0.45	-0.61 -0.73 – -0.49

SDI: sociodemographic index. ASDR: age-standardized DALY rates. ASMR: age-standardized mortality rates. DALYs: disability-adjusted life-years. EAPC: estimate of annual percentage change. UI: uncertainty interval. CI: confidence interval.

In gender-specific analyses (Table 1), the number of deaths for both males and females has increased, but ASMR shows a downward trend. ASMR of males decreased from 7.6 (95% UI: 6.2–9.1) per 100000 population in 1990 to 5.2 (95% UI: 4.1–6.4) per 100000 population in 2019, and EAPC was -1.36 (95% CI: -1.40 – -1.32). Females have similar trends.

From the perspective of the SDI, trends vary across different SDI regions (Table 1, Figure 1C). In high SDI regions, the ASMR significantly decreased from 5.3 per 100000 (95% UI: 4.4–6.3) in 1990 to 1.8 per 100000 (95% UI: 1.5–2.1) in 2019, reflecting a significant decline in IHD mortality related to SHS exposure. In low SDI areas, the mortality decrease was relatively minor, dropping slightly from 5.1 per 100000 (95% UI: 4–6.4) in 1990 to 4.5 per 100000 (95% UI: 3.5–5.6) in 2019.

**Figure 2:**
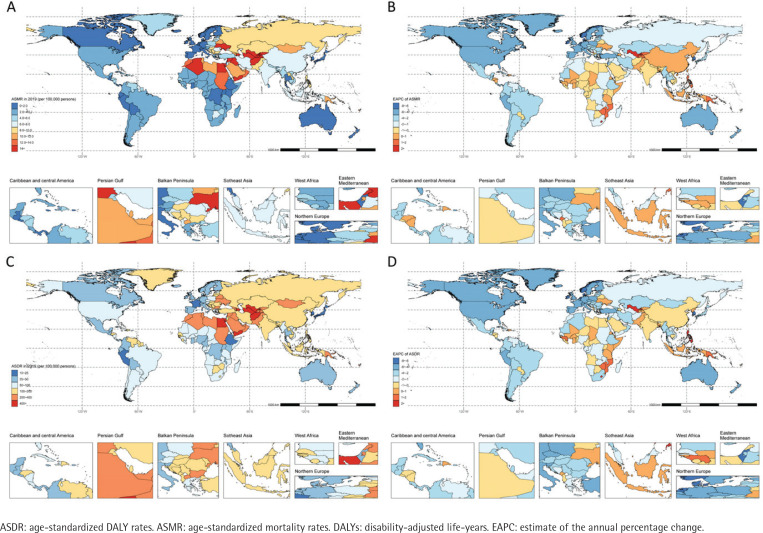
The spatial distribution of ischemic heart disease (A) ASMR, (B) the EAPC of ASMR, (C) ASDR, and (D) the EAPC of ASDR attributable to secondhand smoke in 2019

We also observed noticeable geographical differences in ASMR in 2019 [lowest in France (0.7 per 100000 population, 95% UI: 0.6–0.9) and highest in the Solomon Islands (28.3 per 100000 population, 95% UI: 20.5–37.9)] (Figures 2A and 2B, and Supplementary file Table S1). Overall, 168 countries showed a downward trend, with 36 countries experiencing an increase in ASMR. The highest IHD burden due to SHS in 2019 was observed in East and South Asia, the Middle East, and North Africa, and the lowest in the Andean, Latin American, Australasian, and Oceania regions (Table 1, Figures 2A and 2B).

There were significant differences in the distribution of SHS-related IHD deaths in 2019 among different age groups. The bulk of these deaths occurred in high-middle, middle, and low-middle SDI regions and were primarily concentrated among those aged 65–89 years (Figure 1E, and Supplementary file Table S2). Mortality rates from IHD due to SHS exposure increased with age in all age groups (Figure 1G). The age group of over 60 years accounted for the highest percentage of deaths across every SDI region, and this group’s death rate significantly decreased with time (Supplementary file Figure S1A). In contrast, there were relatively fewer fatalities in age groups under 50 years.

Regarding mortality rates, all SDI regions showed a decreasing trend in mortality rates both globally and regionally; however, there were still notable variations between age groups. Globally, mortality rates decreased between 1990 and 2019. However, the rate of decline was relatively slower for older age groups (Supplementary file Figure S1A). According to an analysis of SDI regions, high SDI areas had the most significant decreases in mortality rates. Meanwhile, low-middle and low SDI regions experienced a slower decline rate (Supplementary file Figure S1B).

### Ischemic heart disease DALYs and ASDR associated with secondhand smoke

Globally, there was a substantial increase in the total number of IHD-related DALYs attributable to SHS, rising from 6.97 million (95% UI: 5.67–8.35) in 1990 to 9.57 million (95% UI: 7.72–11.53) in 2019. From 1990 to 2019, the EAPC of ASDR was -1.43 (95% UI: -1.47 – 1.38) (Table 1, Figures 1B–1D). The results indicate that although the absolute disease burden is increasing with an aging population, the age-standardized disease burden levels are decreasing yearly.

Overall and for both males and females, DALYs and ASDR both increased from 1990 to 2019, but the EAPC of ASDR decreased (males: -1.38; 95% CI: -1.42 – -1.34; females: -1.48; 95% CI: -1.55 – -1.41) (Table 1).

There are significant differences among regions with different levels of SDI. The burden of DALYs is highest in regions with middle SDI, followed by those with high-middle SDI and low-middle SDI. Low-SDI and high-SDI regions exhibit relatively lighter burdens of DALYs. The level of SDI is negatively correlated with ASDR; meanwhile, the higher the level of socioeconomic development, the faster the rate of ASDR decline (Table 1, Figure 1D).

We also observed noticeable geographical differences in ASDR in 2019 (lowest in the Republic of Korea (16.4 per 100000 population, 95% UI: 13–20.4) and highest in the Solomon Islands (827.1 per 100000 population, 95% UI: 600.9–1102.4) (Figures 2C and 2D, and Supplementary file Table S1). Overall, 170 countries showed a downward trend, with 34 countries experiencing an increase in ASMR. The highest IHD burden due to SHS in 2019 was observed in Oceania, North Africa, and the Middle East and Eastern Europe, and the lowest in high-income Asia Pacific, Andean Latin America, and Western Europe regions (Table 1, Figures 2C and 2D).

The burden of DALYs due to IHD caused by SHS is primarily concentrated among individuals around the age of 60 years, particularly within the age group of 50–69 years (Figure 1F, and Supplementary file Table S2). As age increases, the DALYs gradually rise across all age groups, with individuals aged ≥60 years exhibiting significantly higher DALYs rates than other age groups. Overall trends indicate that in most SDI regions, DALYs rates begin to decline after the age of 75 years, possibly due to increased mortality rates among the elderly population. However, DALYs rates in moderate-income countries continue to rise among the elderly (Figure 1H). From 1990 to 2019, there has been a decrease in DALYs rates across most age groups and SDI regions (Supplementary file Figure S2A). Regarding the EAPC of ASDR (Supplementary file Figure S2B), a decreasing trend is observed in most age groups and SDI regions. In terms of age distribution, there is a faster decline in ASDR among individuals aged <65 years, while the decline among the elderly population is relatively slower.

### Global Burden of ischemic heart disease associated with secondhand smoke by sex and age in 2019

**Figure 3:**
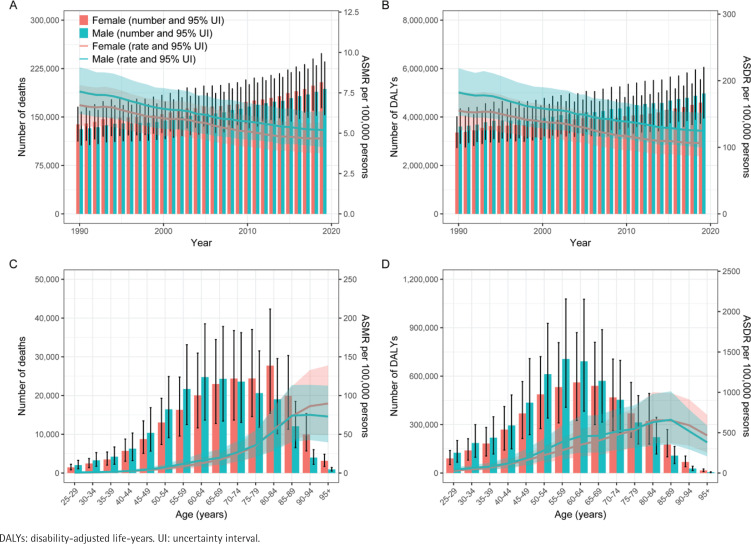
Year-specific numbers and rates of deaths (A) and DALYs (C), and Age-specific numbers and rates of deaths (B) and DALYs (D) of ischemic heart disease attributable to secondhand smoke by sex, in 2019

Both male and female mortality due to SHS-induced IHD has shown an upward trend from 1990 to 2019, with male mortality increasing significantly more than female mortality (Figure 3A). Concurrently, changes in DALYs follow a similar trend to mortality, with the growth rate of DALYs among males surpassing that of females (Figure 3B). In 2019, the burden of death and disease attributable to SHS-induced IHD is primarily concentrated among the elderly population, particularly those aged ≥60 years (Figures 3C and 3D). In terms of gender differences, males generally face a higher risk of mortality compared to females. However, females bear a higher burden of DALYs than males, which may be related to differences in life expectancy between the sexes.

### Association between the SDI values and the ASMR and ASDR of ischemic heart disease

**Figure 4:**
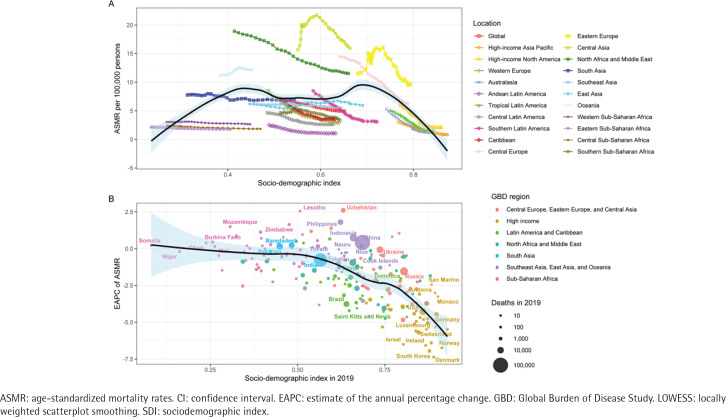
The relationship between ASMR of ischemic heart disease attributed to secondhand smoke in super GBD regions and SDI, 1990 to 2019 (A), and the relationship between EAPC of ASMR and SDI in 2019 (B). Expected trends in all locations were shown as the black line and shaded area (95% CI) with LOWESS methods

**Figure 5:**
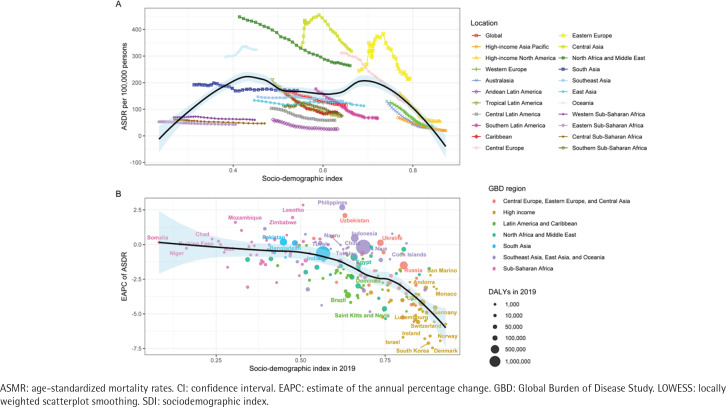
The relationship between ASDR of ischemic heart disease attributed to secondhand smoke in super GBD regions and SDI, 1990 to 2019 (A), and the relationship between EAPC of ASDR and SDI in 2019 (B). Expected trends in all locations were shown as the black line and shaded area (95% CI) with LOWESS methods

The relationship between SHS-induced IHD ASMR and the SDI follows an M-shaped curve (Figure 4A). ASMR tends to be relatively low when the SDI is either low or high; however, it is higher when the index is at a moderate level. The ASMR attributed to SHS-induced IHD shows a decreasing trend in the majority of regions (Figure 4B). The ASDR of IHD caused by SHS exhibits a global downward trend, with variations in the magnitude of decline among different countries and regions, mirroring the pattern observed in ASMR and demonstrating similar characteristics of an M-shaped curve (Figure 5).

## DISCUSSION

This study showed disparities in IHD burden due to SHS globally. The number of deaths and DALYs rates caused by SHS-induced IHD increased, yet the ASMR and ASDR showed a downward trend. The burden of IHD has decreased for both men and women. Only high-SDI countries have seen a significant reduction in the IHD burden, with other SDI category countries experiencing declines but to a less extent. In 2019, the burden of IHD caused by SHS was more significant in Southeast Asia, North Africa, and the Middle East. In contrast, Latin America, Australia, and Oceania regions had a relatively lighter burden. Finally, the burden of IHD caused by SHS is higher in the elderly population.

SHS is a complex mixture of thousands of compounds, including particulate matter released during the combustion of tobacco products and smoke exhaled by smokers^13^. According to the 2019 GBD, SHS caused 1.3 million (95% UI: 1.0–1.6 in millions) deaths globally in 2019^14^. In recent years, growing evidence has shown that exposure to SHS is associated with numerous adverse health outcomes in both children and adults, including respiratory diseases, cardiovascular diseases, kidney diseases, diabetes, neurodevelopmental issues, and myopia in children^15-17^. As highlighted in the literature, even brief and temporary exposure to SHS can impact the development of human atherosclerosis^18^. A conservative estimate from a recent study suggests that SHS exposure increases the risk of IHD by at least 8%^3^. Exposure to SHS increases the risk of IHD through various mechanisms, including endothelial dysfunction, increased oxidative stress, inflammatory responses, heightened blood viscosity, and autonomic nervous system dysregulation^3,6^. Harmful components in SHS, such as nicotine and carbon monoxide, impair vasodilatory function and increase the risk of atherosclerosis; simultaneously, oxidative stress and elevated markers of inflammation (e.g. C-reactive protein) induced by SHS further promote vascular pathology and thrombosis^5,6^. Additionally, SHS raises the risk of IHD by increasing blood viscosity and affects the autonomic nervous system, as evidenced by elevated heart rates and blood pressure^19^. The interplay of these mechanisms underscores the importance of reducing SHS exposure in decreasing the incidence of IHD and enhancing public health^2^.

Despite progress in global public health interventions targeting SHS exposure over recent decades, our findings underscore that SHS continues to pose a considerable threat to global health. Unlike previous research, this study reveals through a more nuanced geographical and age stratification that the burden of IHD attributed to SHS in high-income countries has declined. Conversely, in low-income and middle-income countries, particularly those lacking comprehensive smoking bans, the IHD burden associated with SHS exposure remains alarmingly high. Behind these disparities, cultural, economic, and policy factors played a crucial role^1^. For instance, high-income countries such as France and South Korea might exhibit a lower IHD burden due to stricter public health policies, heightened public health awareness, and better availability of health services^20^. These countries might have enacted effective smoking bans, reducing exposure to SHS in public places and heightened public awareness of the harms of SHS^21,22^.

In contrast, low-income countries and regions like the Solomon Islands might need more effective public health policies and resources to address SHS exposure. These areas face higher tobacco usage rates and lower public health awareness, possibly lacking sufficient medical resources to address health issues caused by SHS exposure^23-25^. Additionally, cultural factors, such as the societal acceptance of tobacco use in specific communities, might affect the extent of SHS exposure and the resulting IHD burden^24,26^. Economic factors, including the level of economic development and the adequacy of public health investments, also largely determine whether a country or region can take adequate measures to reduce SHS exposure and its associated health impacts^24^. Meanwhile, this research highlights the temporal trends in the impact of SHS exposure on IHD over the past 30 years, providing a wealth of refined data to inform the development of more targeted global public health strategies and interventions^27^.

Our research findings underscore the imperative need to reinforce smoking control policies and diminish public exposure to SHS^28^. The comprehensive enforcement of indoor smoking bans and public education campaigns to elevate awareness of the hazards associated with SHS exposure constitutes a pivotal strategy for safeguarding public health and alleviating healthcare burdens^5,29^. Moreover, fostering smoke-free environments and providing readily accessible smoking cessation support services, such as counseling and nicotine replacement therapies, are crucial for facilitating smokers’ quitting efforts^20,30^. This multifaceted approach demands the collaborative involvement of government agencies, community organizations, and the wider public to foster a healthier living environment and reduce the risk of diseases such as IHD^28,31^. In our study, we also observed that the decline in mortality rates among men and the older age groups is slower, and there is a continuous increase in the DALYs rates among the elderly population in middle-income countries. The findings underscore the effective prevention of IHD, which necessitates a comprehensive approach that combines targeted interventions for high-risk populations with extensive public education to enhance cardiovascular health awareness^20,23^. This strategy entails regular health assessments to manage risk factors such as hypertension and high cholesterol, developing personalized lifestyle modification plans, and disseminating the importance of healthy eating, regular physical activity, and smoking cessation across various media channels^4^. Furthermore, governments should enact policies that support healthy lifestyles, including implementing comprehensive smoking bans and providing cardiovascular disease screening services, especially in underserved communities^32,33^.

### Limitations

Our research encompasses certain limitations. Initially, our dataset originates from the GBD 2019, inheriting the typical limitations of GBD, such as inaccuracies in modeling processes that may impact the precision of the data. Additionally, racial and genetic factors may influence susceptibility to SHS exposure, yet GBD 2019 does not capture information on different races^34^. Thirdly, our analysis concentrates on IHD; however, within the GBD study, the categorization of IHD deaths could be inaccurately marked due to the challenges in determining the exact cause. Finally, our study is based on ecological and cross-sectional data from the GBD study, which limits the ability to establish causality between secondhand smoke exposure and ischemic heart disease.

## CONCLUSIONS

Our research utilized data from the GBD 2019 to analyze the burden of IHD from exposure to SHS comprehensively. While the age-standardized burden rates exhibited a declining trend globally, the absolute number of deaths and DALYs attributable to SHS exposure increased substantially from 1990 to 2019. The burden remains a significant public health challenge, particularly in countries with middle and low-middle SDI levels, especially among the elderly population. Our findings advocate for the strengthening of smoking control policies and the implementation of measures to reduce public exposure to SHS, contributing to global efforts to alleviate the burden of IHD.

## Supplementary Material



## Data Availability

The data supporting this research are available from the authors on reasonable request.
