# Supplementary material for: Global, regional, and national burden of ischemic heart disease attributable to secondhand smoke from 1990 to 2019
Source: Tob Induc Dis. 2024 Jul 4;22:10.18332/tid/189771. doi: 10.18332/tid/189771 (PMC11223517; doi:10.18332/tid/189771)
Supplement: Supplementary file 1 [file TID-22-123-s1.pdf]

SUPPLEMENTARY FIGURES

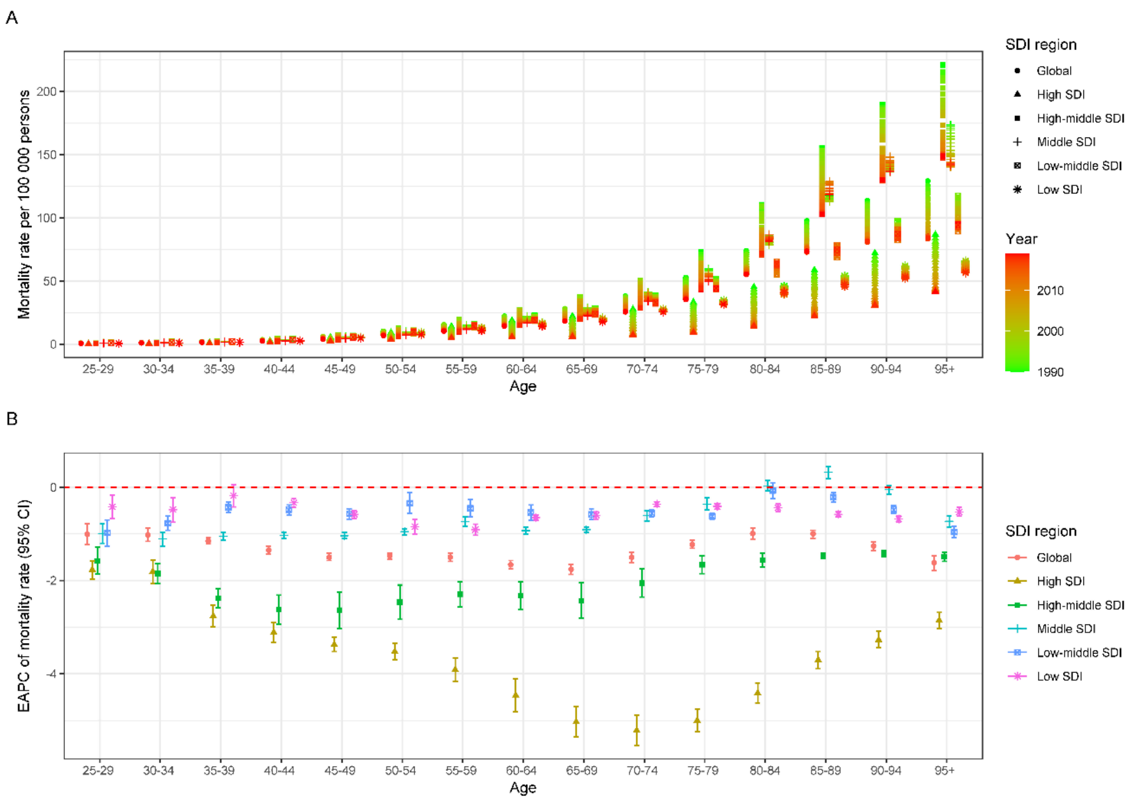

Supplementary Figure 1: The age distribution of (A) age-specific mortality rate and (B). EAPC in age-specific mortality rate attributable to secondhand smoke by SDI region from 1990 to 2019; EAPC: estimated annual percentage change; SDI: Socio-demographic Index.

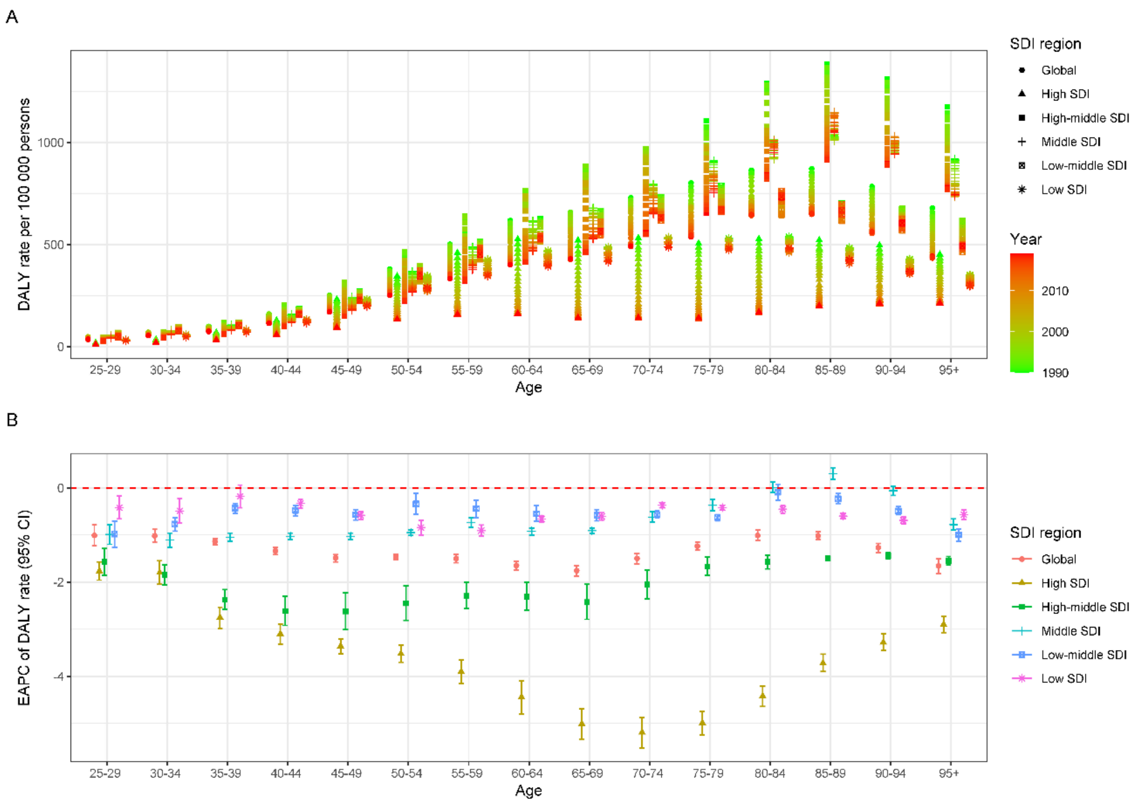

Supplementary Figure 2: The age distribution of (A) age-specific DALYs rate and (B) EAPC in age-specific DALYs rate attributable to secondhand smoke by SDI region from 1990 to 2019. DALYs: disability-adjusted life year; EAPC: estimated annual percentage change; SDI: Socio-demographic Index.

SUPPLEMENTARY TABLES

Supplementary Table 1. Ischemic heart disease burden attributable to secondhand smoke in 1990 and 2019 and its temporal trends from 1990 to 2019 by nation.

**characteristic**	**1990**				**2019**				**EAPC (1990-2019)**	
	**Death cases, n (95% UI)**	**ASMR per 105, n (95% UI)**	**DALYs, n (95% UI)**	**ASDR per 105, n (95% UI)**	**Death cases, n (95% UI)**	**ASMR per 105, n (95% UI)**	**DALYs, n (95% UI)**	**ASDR per 105, n (95% UI)**	**ASMR, n (95% CI)**	**ASDR, n (95% CI)**
Afghanistan	1600.9 (1164.6-2153.8)	22.7 (16.9-29.9)	46333 (33123.4-64451.9)	610.3 (441.6-838.8)	2393.1 (1695.9-3220)	18.6 (13.4-23.9)	76553.4 (52851.4-107442.6)	473.6 (335.8-630.3)	-0.87 (-1.02--0.72)	-1.08 (-1.27--0.89)
Albania	184 (150.9-220.3)	10 (8.1-12)	4076.4 (3344.7-4862.6)	194.8 (160.4-232.4)	329.9 (231.3-438.2)	7.8 (5.5-10.4)	6154.4 (4312.9-8422.8)	149.6 (106.2-203.1)	-0.65 (-0.83--0.47)	-0.73 (-0.92--0.54)

Algeria	2458.8 (1876.2-3111.9)	24.1 (18.8-30.3)	66592.5 (50334-85132.6)	520.9 (401.1-657.9)	4078.5 (3018.7-5364.2)	14.1 (10.5-18.1)	96150.6 (69755.4-128611.3)	277.4 (203-366)	-1.94 (-2.04--1.84)	-2.35 (-2.46--2.24)
American Samoa	2.2 (1.8-2.8)	10 (7.9-12.2)	68.9 (53.6-85.4)	255.1 (200.5-315)	4.3 (3.3-5.4)	9.1 (6.9-11.4)	121 (90.1-155.5)	234.7 (176.1-301.3)	-0.37 (-0.45--0.3)	-0.35 (-0.43--0.28)
Andorra	1.4 (1-2)	2.7 (2-3.9)	37 (26.6-53.8)	64.3 (46.6-92.7)	1.8 (1.3-2.5)	1.2 (0.9-1.7)	40.5 (27.9-55.6)	29.3 (20-40.3)	-3 (-3.17--2.83)	-3.05 (-3.21--2.89)
Angola	120.2 (83.3-160.5)	3 (2.1-4)	3829.8 (2620.5-5163.2)	79.4 (55-105.8)	299.7 (208.1-422.8)	2.8 (2-3.8)	9146.2 (6321.5-13158.6)	67.7 (47.2-95.2)	-0.34 (-0.42--0.26)	-0.63 (-0.72--0.55)
Antigua and Barbuda	1.9 (1.5-2.4)	3.6 (2.9-4.5)	44.4 (35.3-54.9)	88.4 (70.1-110.1)	1.9 (1.5-2.5)	2 (1.5-2.6)	45.8 (34.6-59.3)	43.8 (33.2-56.2)	-2.32 (-2.53--2.11)	-2.75 (-2.97--2.53)

Argentina	2717.5 (2175.7-3276.8)	8.9 (7.2-10.7)	60499.9 (48304.7-72842.8)	189.1 (152.1-226.7)	1844.9 (1463.7-2254.3)	3.4 (2.7-4.1)	38489.2 (30556.2-47056.4)	73.5 (58.3-90.2)	-3.46 (-3.69--3.22)	-3.46 (-3.66--3.26)
Armenia	441.2 (361.6-522.4)	19.2 (15.7-22.9)	9492.5 (7754.7-11308.2)	361.8 (297.2-428.6)	470.5 (360.2-598.6)	11.7 (9-14.7)	9076 (6966-11584.7)	222.5 (171.7-280.2)	-2.53 (-2.77--2.29)	-2.34 (-2.55--2.14)
Australia	1006.4 (791-1242.2)	5.3 (4.2-6.5)	23960.3 (19137.5-29373.6)	126.2 (101.6-154.8)	466.2 (368.1-575.1)	1.1 (0.9-1.4)	9998.3 (7995.6-12282.3)	27.8 (22.4-34.4)	-5.59 (-5.86--5.32)	-5.42 (-5.69--5.15)
Austria	583.3 (462.3-705.1)	5.1 (4.1-6.1)	13192.4 (10649-15801.8)	123.8 (100.2-148.2)	454.8 (363.3-551)	2.4 (2-2.9)	8258.9 (6668.6-9897.3)	51.4 (41.7-61.4)	-3.09 (-3.31--2.88)	-3.61 (-3.85--3.37)
Azerbaijan	943.4 (769.7-1125)	20.1 (16.4-24)	22594.1 (18508.7-27153)	438.7 (361-524.9)	1660.9 (1289.4-2080.9)	23.3 (18.3-28.8)	38095.7 (29569-47712.8)	425.7 (332.9-529.2)	0.19 (0-0.38)	-0.58 (-0.78--0.37)

Bahamas	6.9 (5.5-8.4)	4.5 (3.6-5.5)	198.2 (155.3-243.4)	115.3 (90.4-141.6)	10.6 (8.1-14)	2.7 (2.1-3.5)	299.4 (223-397.8)	70.1 (52.6-92.4)	-2.02 (-2.21--1.82)	-1.93 (-2.12--1.74)
Bahrain	35.9 (28.5-44.4)	22.1 (17.8-27.2)	1083.5 (856.5-1345.5)	489.7 (390-606)	50.6 (37.1-66.9)	6.4 (4.8-8.3)	1483.5 (1089.7-1973.3)	126.8 (94.5-165.5)	-4.75 (-5.01--4.49)	-5.35 (-5.63--5.06)
Bangladesh	2791.7 (2113.4-3565)	6.1 (4.6-7.7)	81324.4 (59530.1-105394.2)	151.7 (112.8-195.7)	6943.5 (4991.2-9433.7)	5.5 (4-7.4)	177778.4 (127468-240154.4)	129.8 (92.6-176)	0.22 (-0.06-0.5)	0.1 (-0.18-0.38)
Barbados	7.9 (6.3-9.8)	2.8 (2.2-3.5)	174.6 (138.9-216.3)	66.8 (53.4-82.5)	6.1 (4.5-8)	1.3 (0.9-1.6)	141.4 (102.2-184.8)	30.5 (22.3-39.9)	-3.29 (-3.55--3.03)	-3.25 (-3.49--3.01)
Belarus	1344.6 (1087.9-1606.4)	10.8 (8.7-12.9)	28903.6 (23217.6-34488.5)	227.6 (184-271.1)	1668.9 (1249.8-2159.5)	10.4 (7.8-13.5)	33583.6 (24905.3-44360.3)	217.8 (162.4-288)	-0.46 (-0.83--0.09)	-0.56 (-1.03--0.09)

Belgium	680.4 (551.3-827.1)	4.6 (3.8-5.5)	15694.7 (12760.6-18890.7)	113.4 (92.5-135.5)	292.3 (234.2-352.1)	1.3 (1.1-1.6)	6052.4 (4918.8-7318.2)	31.8 (25.9-38.5)	-4.68 (-4.84--4.52)	-4.69 (-4.84--4.54)
Belize	3.4 (2.7-4.2)	3.7 (2.9-4.5)	84.5 (66.3-103.7)	88.1 (69-108)	6.5 (5-8.2)	2.4 (1.8-3)	179.7 (135.1-230.4)	58 (43.8-73.6)	-2.08 (-2.5--1.66)	-2.08 (-2.5--1.67)
Benin	56.5 (41.6-73.1)	2.9 (2.2-3.8)	1465.6 (1072.1-1920.4)	69.3 (51.1-90.5)	113.4 (82.4-155)	2.4 (1.8-3.2)	3211.4 (2243.7-4508.3)	57.5 (41.5-80)	-0.79 (-0.88--0.7)	-0.75 (-0.85--0.65)
Bermuda	4.5 (3.5-5.6)	7.4 (5.9-9.2)	108 (85-134)	169.4 (133.2-210.8)	2.7 (2.1-3.5)	2.1 (1.6-2.7)	53.5 (40-69.7)	44.5 (33-57.7)	-4.64 (-5.06--4.22)	-4.82 (-5.26--4.37)
Bhutan	10.5 (7-14.5)	4.2 (2.9-5.7)	339.9 (218.3-477.8)	111.3 (73.6-154.6)	20.5 (14.6-27.7)	3.7 (2.7-5)	562 (395.1-779.5)	90.8 (64.6-125.3)	-0.58 (-0.64--0.52)	-0.92 (-0.99--0.85)

Bolivia (Plurinational State of)	87 (58.1-125)	2.8 (1.9-4)	2407.6 (1571.5-3514)	68 (45.1-97.9)	129.1 (80.8-189.3)	1.5 (1-2.2)	3276.8 (2006.5-4933)	35.3 (21.8-52.6)	-2.27 (-2.65--1.89)	-2.44 (-2.78--2.1)
Bosnia and Herzegovina	389.1 (316-468)	10.8 (8.8-13)	9616.7 (7725.3-11507.6)	229.1 (186.5-274.6)	461 (333.9-606.2)	8.1 (6-10.6)	8822.8 (6375.7-11618)	155 (112.9-203.9)	-1.41 (-1.56--1.26)	-1.9 (-2.07--1.74)
Botswana	35.5 (25.4-47.9)	6.9 (5.1-9.2)	945.7 (666.1-1307.4)	156.6 (112.4-213.2)	81.9 (53.9-116.7)	6.6 (4.4-9.4)	2266.1 (1451.5-3319.9)	148.5 (97.9-212.6)	-0.49 (-0.85--0.13)	-0.61 (-1.06--0.15)
Brazil	7489.1 (6125.4-8963.3)	8.7 (7.1-10.4)	211492.5 (173217.3-253398.6)	214.3 (175.1-256.7)	6934.1 (5570.8-8478.5)	2.9 (2.4-3.6)	182586.9 (145761.4-222729.1)	74.6 (59.9-91.1)	-3.76 (-3.93--3.58)	-3.65 (-3.81--3.49)
Brunei Darussalam	6.9 (5.5-8.6)	6.9 (5.5-8.5)	228 (178.2-288.3)	169.9 (135.2-212.2)	9.6 (7.5-12)	3.2 (2.5-3.9)	308 (236.7-391.8)	78.3 (61.4-98.1)	-2.67 (-2.86--2.48)	-2.71 (-2.97--2.45)

Bulgaria	1904.6 (1556.6-2283)	17.6 (14.6-20.9)	42586 (34869.6-51010.1)	362.7 (301.6-431.8)	1374.9 (1007.3-1794.1)	9.8 (7.2-12.8)	27527.9 (20126.5-36281.6)	216.8 (158.3-286.1)	-3.03 (-3.47--2.58)	-2.76 (-3.22--2.31)
Burkina Faso	123.3 (87.8-163)	3.1 (2.2-4)	3332 (2375.6-4440)	71.5 (51.2-94.4)	289.2 (209.8-383.6)	3.4 (2.5-4.5)	7763.3 (5550.9-10418.9)	77.3 (55.8-102.5)	0.45 (0.3-0.61)	0.24 (0.11-0.37)
Burundi	55.8 (37.8-78.4)	2.3 (1.6-3.3)	1720.8 (1136.5-2497.7)	64.7 (43.1-93)	79 (53-114.4)	1.6 (1.1-2.3)	2537.5 (1697.4-3687.2)	44.1 (29.5-64)	-1.47 (-1.61--1.33)	-1.64 (-1.78--1.49)
Cabo Verde	7.5 (5.7-9.6)	3.2 (2.5-4.1)	156.1 (120.3-198.4)	70 (53.6-88.9)	13.3 (10-16.9)	3.2 (2.4-4.1)	294.6 (220.2-378)	67.1 (50.1-85.7)	-0.56 (-0.91--0.21)	-0.63 (-0.88--0.37)
Cambodia	260.6 (193.8-351.2)	6 (4.5-8)	7740.8 (5748-10728.3)	150.8 (112-202.9)	607.4 (446.8-806.9)	5.5 (4.1-7.3)	16003.3 (11521.9-21577.5)	125.4 (91.7-168.2)	-0.44 (-0.54--0.34)	-0.81 (-0.92--0.71)

Cameroon	93.4 (66.8-126.8)	2.3 (1.7-3.1)	2508.6 (1750.9-3483)	52.2 (36.9-70.8)	239.4 (165.1-340.8)	2.1 (1.5-2.9)	6947.9 (4616.2-10099.9)	50 (34-71.3)	-0.4 (-0.6--0.2)	-0.16 (-0.42-0.09)
Canada	1653.2 (1334.1-2000.4)	5.2 (4.2-6.3)	39815 (32023.3-48307.6)	127.1 (102-154.7)	946 (755-1146.4)	1.4 (1.1-1.7)	20683.2 (16588.4-25525.6)	34.6 (27.9-42.2)	-5.05 (-5.4--4.7)	-4.92 (-5.26--4.57)
Central African Republic	43 (30-58.6)	3.5 (2.6-4.8)	1362.3 (942.3-1926.4)	96.7 (68.5-132.4)	70.7 (48-100)	3.1 (2.2-4.4)	2302.4 (1531-3374.2)	83.7 (57-117.6)	-0.46 (-0.58--0.34)	-0.56 (-0.69--0.43)
Chad	71.3 (50.9-94.2)	2.7 (1.9-3.5)	1798.7 (1286-2399.4)	61.4 (44-80.8)	151.3 (108.9-204.2)	2.9 (2.1-3.9)	4188.4 (2938.4-5745.8)	66.7 (47.6-91)	0.4 (0.32-0.47)	0.4 (0.3-0.51)
Chile	665.6 (529.1-811.2)	7.5 (6-9)	13671.5 (10939.1-16644.4)	138.9 (112-168.9)	571.5 (446.4-699.2)	2.4 (1.9-2.9)	12059.7 (9545.3-14901.5)	50.7 (40.2-62.4)	-3.84 (-4.08--3.6)	-3.31 (-3.49--3.12)

China	44404.9 (34407.5-54685.6)	6.2 (4.9-7.5)	1177170.9 (918073.8-1445583.8)	133.7 (104.4-163.6)	105995 (82606.1-132989.2)	6 (4.7-7.5)	2202888.6 (1708667.7-2777346.3)	113.6 (88.4-141.8)	0.42 (0.18-0.65)	-0.19 (-0.36--0.03)
Colombia	868.2 (683.5-1061.5)	5.2 (4.1-6.4)	22490.2 (17679-27510.8)	118.8 (93.7-145.6)	1208.4 (837.3-1640.2)	2.2 (1.6-3.1)	25446.8 (17487.8-35180.4)	48.2 (33-66.9)	-3.16 (-3.33--2.98)	-3.37 (-3.57--3.17)
Comoros	8.5 (4.9-12)	4.1 (2.5-5.7)	223.8 (110.9-326.1)	95.9 (50.4-137.4)	17.1 (11.5-24.1)	3.7 (2.5-5.1)	449 (293.9-644.8)	86.2 (56.4-123)	-0.51 (-0.64--0.39)	-0.56 (-0.73--0.39)
Congo	41.9 (30.2-56.6)	3.9 (2.9-5.3)	1275.8 (908.7-1744.7)	104.2 (74.5-141.3)	77.8 (50.6-114.8)	3 (2-4.3)	2330.6 (1479.8-3546.8)	74.3 (48.3-110.3)	-0.82 (-0.97--0.68)	-1.17 (-1.31--1.02)
Cook Islands	1.1 (0.8-1.4)	8.7 (6.6-11.3)	30.9 (22.8-41.4)	224.9 (167.2-297.7)	1.5 (1.1-2)	6.3 (4.8-8.2)	37.9 (27.9-50.6)	161.6 (117.3-215.7)	-1.12 (-1.31--0.92)	-1.06 (-1.29--0.83)

Costa Rica	91.2 (72-112.1)	5.4 (4.3-6.6)	2141.5 (1707.9-2626.9)	118 (93.8-144.2)	120.3 (85.7-162.6)	2.3 (1.6-3.1)	2848.3 (2019.6-3917.2)	54.4 (38.7-74.6)	-3.4 (-3.77--3.03)	-3.12 (-3.48--2.76)
C 么 te d'Ivoire	186.6 (137.2-246.5)	5.2 (3.9-6.7)	5511.8 (4016.7-7385.5)	118.4 (87.2-155.9)	443.2 (309.1-600)	4.6 (3.3-6.1)	12616.9 (8624.4-17500.3)	103.1 (71.5-140.3)	-0.62 (-0.95--0.3)	-0.75 (-1.11--0.39)
Croatia	990.7 (809.4-1191)	16.8 (13.9-20.2)	21167.7 (17293.9-25409.9)	337.6 (279.2-403.2)	634.9 (456.9-830.2)	7.1 (5.2-9.2)	11029.4 (7972.4-14402.5)	135.2 (98.5-177.8)	-2.53 (-2.71--2.34)	-2.84 (-2.99--2.7)
Cuba	928.4 (750.3-1121.7)	9.2 (7.4-11.1)	21379 (17331.4-25658.6)	207.9 (168.6-250)	715.1 (535.8-930.7)	3.7 (2.8-4.8)	15130.3 (11162.9-19881.6)	82.6 (60.9-108.6)	-3.67 (-3.96--3.38)	-3.68 (-3.96--3.39)
Cyprus	55.5 (44.3-68.1)	7.8 (6.3-9.6)	1358.5 (1088.2-1662.9)	174.3 (139.1-214.3)	54.4 (42.8-68.1)	3.1 (2.4-3.8)	1278 (1000.4-1598.4)	71.1 (56-88.7)	-3.69 (-3.92--3.46)	-3.57 (-3.82--3.31)

Czechia	1844.4 (1468.8-2271)	13.7 (11-16.6)	41222 (33085.7-50566)	309.5 (250.7-376.8)	1031 (762.5-1331.7)	4.9 (3.6-6.2)	18267.5 (13312.6-23558.8)	93.2 (67.8-120.1)	-3.75 (-3.92--3.59)	-4.34 (-4.52--4.15)
Democratic People's Republic of Korea	967.7 (694.4-1268.5)	6.9 (5-9.1)	26471.6 (18574.9-35766.8)	156.3 (112.3-207.7)	2152.5 (1633.3-2820.2)	7.1 (5.4-9.3)	51344.5 (38223.3-68830.5)	159.5 (118.6-211.7)	0.16 (-0.03-0.35)	0.1 (-0.06-0.27)
Democratic Republic of the Congo	303.2 (204.2-437.1)	2 (1.4-2.8)	9038.1 (6057-13143.9)	49.6 (33.4-71.7)	495.9 (310.3-732.6)	1.4 (0.9-2.1)	14995.1 (9403.9-22559.1)	35.2 (22-52.5)	-1.41 (-1.54--1.28)	-1.38 (-1.5--1.25)
Denmark	631.8 (508.5-764.8)	8 (6.5-9.6)	13585.1 (11035.8-16438.3)	185.9 (150.7-224.5)	138.9 (109.8-170.2)	1.2 (1-1.5)	2704 (2138.8-3305.9)	26.9 (21.5-32.9)	-7.36 (-7.75--6.96)	-7.45 (-7.84--7.07)
Djibouti	4.9 (3.5-6.8)	4.2 (3.1-5.7)	145.2 (100.9-203.5)	90.8 (65.3-124.2)	25.3 (16.5-37.3)	5.1 (3.4-7.2)	705.6 (443.5-1064)	107 (69.8-157.7)	0.78 (0.6-0.97)	0.68 (0.49-0.87)

Dominica	2.4 (1.9-3)	3.4 (2.7-4.2)	51.5 (40.1-64.9)	77.3 (60.4-97.4)	1.8 (1.3-2.3)	1.9 (1.4-2.5)	38.2 (27.6-50.2)	43.3 (31.1-56.9)	-2.18 (-2.45--1.9)	-2.26 (-2.54--1.98)
Dominican Republic	170.1 (135.8-208.1)	4.6 (3.7-5.6)	4982.8 (3946.6-6087.4)	116.2 (92.7-142.3)	459.6 (325.2-630.8)	4.9 (3.5-6.7)	12542 (8663-17559.8)	126.8 (88-177.1)	0.66 (0.39-0.93)	0.57 (0.29-0.86)
Ecuador	123.3 (97.8-152.2)	2.4 (1.9-2.9)	3412.2 (2696.7-4231.5)	56.7 (45-69.9)	192.5 (134.4-263.7)	1.3 (0.9-1.8)	5016 (3401.5-6932.5)	31.8 (21.9-44.1)	-2.11 (-2.37--1.85)	-2.22 (-2.44--2)
Egypt	6925.9 (5555.7-8405.3)	25.2 (20.3-30.5)	194335.5 (156780.6-236333)	598.6 (482.7-723.6)	11214.6 (7875.4-15178)	18.5 (13-24.6)	313083.2 (216335.6-434501)	440.1 (309.1-600.8)	-0.94 (-1.03--0.84)	-0.91 (-1--0.83)
El Salvador	69.9 (55.1-85.9)	2.3 (1.8-2.9)	1996.1 (1573.5-2463)	61.4 (48.4-76.1)	119.8 (82.3-163.5)	2 (1.4-2.7)	2806.6 (1920-3876.7)	47.6 (32.5-66.1)	-0.64 (-0.82--0.46)	-0.96 (-1.2--0.73)

Equatorial Guinea	7.5 (5-10.7)	3.8 (2.5-5.5)	226.5 (148.7-320.8)	100.5 (66.7-142.3)	9.3 (5.9-14.2)	2.1 (1.4-3.1)	254.1 (154-406.8)	46.4 (29-71.1)	-2.53 (-2.83--2.22)	-3.2 (-3.58--2.83)
Eritrea	21 (13.5-31.5)	2 (1.3-2.8)	709.2 (443.9-1068.7)	54.1 (34.9-80.2)	65.2 (44.3-92.8)	2.4 (1.7-3.3)	2121.4 (1433.6-3130.1)	61.7 (41.9-87.6)	0.71 (0.6-0.81)	0.5 (0.39-0.61)
Estonia	251.2 (201.1-307.9)	12.6 (10.2-15.3)	5411.3 (4321.5-6588.7)	269 (217.4-328)	108.8 (78.6-153.9)	3.8 (2.8-5.4)	1859.9 (1333.5-2596.4)	75.4 (54.1-105.5)	-5.11 (-5.58--4.65)	-5.56 (-6.09--5.04)
Eswatini	7.6 (5.4-10.2)	2.8 (2-3.7)	212.1 (148.9-292.6)	65.4 (46.2-88.4)	13.5 (8.8-19.5)	2.5 (1.7-3.5)	392.4 (248.4-578.8)	60.3 (39-87.7)	-0.02 (-0.32-0.29)	0.17 (-0.23-0.58)
Ethiopia	357.1 (237.6-508.3)	1.6 (1.1-2.3)	11883 (7828.9-17189.7)	47.7 (31.5-68.9)	361.3 (231.7-505.8)	0.9 (0.6-1.2)	10865.3 (6872.9-15254.7)	21.8 (13.8-30.4)	-2.54 (-2.69--2.38)	-3.07 (-3.23--2.9)

Fiji	54.9 (41.1-72)	14.9 (11.3-19.3)	1749.5 (1305.9-2304.3)	390.7 (292.7-512.7)	88.1 (62.4-118.2)	12.2 (8.7-16.1)	2573.1 (1773.3-3485.2)	308.2 (217.5-412.8)	-0.75 (-0.88--0.61)	-0.84 (-0.98--0.7)
Finland	368 (292.3-455.6)	5.3 (4.3-6.5)	8914.3 (7087.6-11017.8)	133.8 (106.7-165.6)	194.4 (151-241)	1.6 (1.2-2)	3617.6 (2782.2-4485.4)	35.5 (27.3-44.2)	-4.26 (-4.44--4.08)	-4.64 (-4.83--4.45)
France	1851 (1504.9-2236.9)	2.4 (1.9-2.8)	44484.8 (36033.7-53773.4)	61.1 (49.6-73.5)	946.7 (735.8-1161.5)	0.7 (0.6-0.9)	20315.1 (16331.7-24592)	19.6 (15.7-23.8)	-4.42 (-4.65--4.19)	-4.3 (-4.52--4.07)
Gabon	18.9 (13.4-25.4)	3.5 (2.6-4.7)	516.1 (358.3-705.3)	87 (61.2-117.5)	26.9 (18.4-37.1)	2.7 (1.8-3.7)	746.1 (494.7-1049.6)	63.6 (43.1-87.9)	-0.96 (-1.13--0.8)	-1.09 (-1.27--0.9)
Gambia	18 (12.9-24.1)	5.9 (4.4-7.8)	458.8 (319.9-629.7)	124.1 (87.9-166.8)	55.8 (40.1-74.3)	6.3 (4.6-8.3)	1392.5 (965.2-1929)	137.6 (97.7-186.2)	0.2 (0.05-0.35)	0.3 (0.12-0.49)

Georgia	1225.2 (991.7-1480.1)	21.7 (17.7-25.8)	28211.1 (22746.9-34043.6)	463 (375.5-552.5)	720.3 (556.5-927)	11.5 (9-14.9)	13977 (10716.5-18081.7)	243.9 (186.5-313.1)	-2.73 (-3.01--2.44)	-2.93 (-3.25--2.6)
Germany	6350.9 (5152.2-7752.2)	5.2 (4.2-6.3)	154539.4 (124080-189398.8)	134.7 (109-164.9)	2727.8 (2198.4-3334.8)	1.5 (1.2-1.8)	57832.1 (46417.9-70839.6)	38 (30.4-46.7)	-4.46 (-4.74--4.18)	-4.54 (-4.8--4.28)
Ghana	106.6 (77.1-144.2)	1.7 (1.3-2.3)	3308.5 (2345.5-4589.6)	44.1 (31.9-59.9)	285.2 (206.2-383.3)	1.8 (1.3-2.4)	8432.5 (5883.7-11497.5)	44.6 (32.1-60.3)	0.55 (0.42-0.68)	0.4 (0.26-0.54)
Greece	986.6 (799.7-1182.4)	6.7 (5.5-7.9)	22444.5 (18206.7-26800.1)	156 (127.1-186)	838.9 (667.8-1026.6)	3.7 (3-4.5)	17115.7 (13752.3-20794.6)	93.6 (75.4-113.7)	-2.19 (-2.39--1.99)	-1.82 (-2.01--1.64)
Greenland	4.6 (3.7-5.7)	13.1 (10.5-15.9)	142.1 (109.8-178.8)	338 (268.1-418.2)	3.2 (2.2-4.1)	4.5 (3.3-5.8)	85.8 (60.3-113.4)	112.6 (80.7-147.4)	-4.12 (-4.32--3.92)	-4.17 (-4.35--3.99)

Grenada	3.5 (2.8-4.4)	4.9 (3.8-6.1)	79.2 (61.6-99.3)	119.8 (93.7-150.3)	2.8 (2.1-3.5)	2.6 (1.9-3.2)	69.3 (52.1-90.5)	59.5 (45-76.7)	-2.25 (-2.58--1.93)	-2.37 (-2.75--1.99)
Guam	8.4 (6.5-10.5)	12.3 (9.6-15.4)	237.2 (182.1-298.2)	277.1 (216.2-346.3)	20.5 (15.4-26.2)	10.8 (8.2-13.8)	540 (407.5-689.7)	283 (214.5-363.2)	-0.47 (-0.72--0.22)	0.09 (-0.08-0.27)
Guatemala	123.6 (95-155.8)	3.6 (2.8-4.5)	3701.7 (2783.3-4716.9)	87.6 (66.6-110.5)	205.6 (142.1-277.8)	2 (1.4-2.6)	5340.9 (3685.9-7341.1)	44.2 (30.4-59.9)	-2.33 (-2.73--1.93)	-2.57 (-3.01--2.12)
Guinea	95.1 (68.4-127)	3.1 (2.2-4.1)	2409.9 (1712.2-3248.8)	70.1 (50.3-93.5)	185.2 (133.1-250.3)	3.5 (2.5-4.7)	4988.6 (3514.1-6815.8)	82.8 (58.5-112.7)	0.87 (0.72-1.02)	1.02 (0.87-1.16)
Guinea-Bissau	19.3 (13.3-27)	4.9 (3.5-6.8)	560.4 (380.8-805.4)	123.7 (86-175.5)	33.6 (23.8-46.2)	4.7 (3.4-6.5)	1026.2 (709-1448.1)	118.5 (83.9-163.2)	0.2 (0.09-0.31)	0.18 (0.06-0.29)

Guyana	41.1 (31.7-51)	11 (8.6-13.7)	1169.8 (898.2-1469.8)	276.4 (212.7-348.8)	38.1 (26.4-52.1)	6.2 (4.4-8.3)	1054.4 (719.7-1461.7)	153.6 (106.3-209.7)	-2.03 (-2.18--1.88)	-2.06 (-2.18--1.94)
Haiti	181.1 (130.9-241.2)	5.6 (4.1-7.5)	5393.1 (3873.3-7224.9)	146.2 (105.6-195.5)	248.6 (158.8-367.2)	3.6 (2.4-5.3)	7288.1 (4577.3-10910.2)	90.5 (57.6-134.5)	-1.43 (-1.77--1.08)	-1.52 (-1.9--1.15)
Honduras	91.4 (68.7-121.3)	4.5 (3.3-6.1)	2558.7 (1959.6-3338.7)	110.3 (84.2-145.1)	278.7 (208.3-369.2)	5 (3.8-6.6)	6614.3 (4816.3-8880.9)	106.2 (78.3-140.1)	0.49 (0.31-0.67)	-0.06 (-0.21-0.09)
Hungary	2309.9 (1898.5-2765.6)	16.6 (13.7-19.6)	53062.8 (43354.5-63745.8)	379.8 (312.1-454)	1609.2 (1188.6-2053.7)	8.2 (6-10.4)	29499.7 (21883.4-38089.6)	164.5 (121.8-212.1)	-2.61 (-2.73--2.49)	-3.14 (-3.26--3.01)
Iceland	20.8 (17.1-25.3)	7.4 (6.1-8.9)	471.1 (387.1-568.2)	175.2 (144.4-211.4)	9.4 (7.5-11.6)	1.7 (1.4-2.1)	199.8 (160-244)	40.2 (32.2-49)	-5.48 (-5.8--5.15)	-5.5 (-5.88--5.12)

India	34011.1 (26206.2-41973.6)	8.1 (6.3-9.9)	1026099.3 (796933.2-1275724.4)	197 (152.5-241.7)	74999 (57956.5-94664.1)	6.8 (5.3-8.6)	2065177.1 (1581007.7-2593752.7)	168.6 (129.4-211.9)	-0.77 (-0.92--0.62)	-0.65 (-0.81--0.49)
Indonesia	5832 (4487.1-7307.2)	6 (4.6-7.5)	180874 (139396.4-225405.7)	154.1 (119-192.6)	14175.2 (10801.2-17821)	7 (5.4-8.7)	398262.5 (303792.3-508804)	165.5 (127-209.6)	0.74 (0.67-0.81)	0.46 (0.39-0.54)
Iran (Islamic Republic of)	3307.5 (2604-4029)	14 (11.2-17)	93133.1 (73392.9-113515.8)	322.5 (255.8-391.4)	5084.4 (4020.9-6120.3)	7.4 (5.8-9)	121526.3 (97226.1-147183.9)	157.2 (125.9-190.8)	-2.69 (-2.96--2.43)	-2.98 (-3.24--2.71)
Iraq	1555.7 (1216-1969.4)	20.8 (16.4-26.4)	40818 (31752.2-51551.8)	492.4 (382.9-622.7)	3286.5 (2422.7-4260)	15.8 (11.8-19.9)	84939.8 (60690.2-111911.5)	345.7 (250.4-450.2)	-1.22 (-1.33--1.11)	-1.55 (-1.7--1.4)
Ireland	385.7 (312.4-461.6)	9.7 (7.9-11.5)	8962.4 (7296.4-10714.2)	232.7 (189.3-277.4)	132 (103.9-163.2)	1.8 (1.4-2.2)	2779.1 (2182.8-3391)	39.5 (31-48.1)	-6.57 (-6.87--6.27)	-6.79 (-7.08--6.5)

Israel	270.2 (218.6-324.1)	5.8 (4.7-6.9)	6521.2 (5340.4-7761.3)	141.8 (116.1-168.7)	126.2 (98.7-154.3)	1.1 (0.9-1.3)	2674 (2118.2-3248.1)	25.1 (19.8-30.6)	-6.5 (-6.86--6.13)	-6.69 (-7.07--6.3)
Italy	4022.7 (3243.7-4835.7)	4.7 (3.8-5.6)	91437 (74353.5-109708.6)	111.2 (90.8-133.1)	2311 (1819.6-2829.9)	1.6 (1.3-1.9)	43254.9 (35052.6-52424.5)	37 (30.2-44.3)	-3.93 (-4.07--3.79)	-4.01 (-4.15--3.87)
Jamaica	48 (38.6-57.5)	2.7 (2.2-3.2)	1039 (837.3-1249.9)	60.2 (48.4-72.4)	60.1 (43.6-78.5)	2 (1.4-2.6)	1394.8 (991.9-1845.8)	46.6 (33.1-61.6)	-1.29 (-1.71--0.87)	-1.04 (-1.49--0.58)
Japan	4105.3 (3278.8-4980.8)	2.5 (2-3.1)	89822.3 (73339.8-108457.4)	53.7 (43.8-64.8)	3013.1 (2282.8-3782.9)	0.9 (0.7-1.1)	53681.5 (42621.8-65630.4)	21.4 (17.2-26)	-3.72 (-3.88--3.56)	-3.12 (-3.2--3.03)
Jordan	187.2 (146.9-230.3)	15.2 (12-18.7)	5196.5 (4083.1-6466.4)	347.4 (272.8-428.5)	391.8 (301.9-496.9)	6.4 (5-8.1)	10686.1 (8263.7-13666.4)	144.9 (111.9-183.9)	-3.81 (-4.18--3.44)	-3.88 (-4.26--3.5)

Kazakhstan	1628.2 (1321.5-1961.5)	14.1 (11.4-17)	38075.6 (30732.6-46185.8)	297.3 (242-358.7)	1395.7 (1078-1788.1)	9.4 (7.4-12)	30960 (23564.4-39663.8)	181.1 (139-231.1)	-2.12 (-2.84--1.38)	-2.62 (-3.48--1.75)
Kenya	117.2 (86.8-154.9)	1.6 (1.2-2.1)	2994.3 (2197.7-3997.1)	34.3 (25.4-45.6)	322.2 (223.3-438.2)	1.6 (1.1-2.1)	9205.4 (6355.8-12705.1)	36.3 (25.3-49.2)	-0.09 (-0.27-0.09)	0.22 (-0.06-0.5)
Kiribati	8.5 (6.3-11.3)	21 (15.7-27.1)	295.8 (214.9-393.3)	639.9 (470.5-839.6)	14.6 (10.4-19.5)	19.5 (14.1-25.5)	497.9 (358.1-675.9)	566.1 (409.3-760.3)	-0.43 (-0.59--0.28)	-0.62 (-0.78--0.46)
Kuwait	71.6 (58.7-85.7)	11.5 (9.3-13.8)	2254.1 (1833.6-2713.2)	273.6 (222.4-329.8)	165 (127.3-211.9)	5.9 (4.6-7.6)	5138.5 (3884.5-6669.5)	141.5 (108.3-181.9)	-2.19 (-2.51--1.86)	-2.17 (-2.53--1.81)
Kyrgyzstan	380.8 (308.1-457.4)	13.1 (10.6-15.6)	8491.4 (6944.5-10081.4)	277.8 (227.6-330.1)	646.4 (513.2-803.9)	17 (13.4-21.3)	13354.5 (10537.6-16736.9)	306.3 (240.7-384.5)	1.39 (0.92-1.86)	0.59 (0.15-1.02)

Lao People's Democratic Republic	207.7 (149.4-284.8)	10.9 (7.8-14.7)	5804.5 (4110.1-8079.2)	258.8 (185.1-357.4)	341 (251.2-443.3)	8.7 (6.6-11)	9168.6 (6637-12099.2)	191.3 (141.3-248.3)	-1.01 (-1.13--0.89)	-1.24 (-1.37--1.1)
Latvia	437 (349.9-531.9)	12.4 (10-15.1)	9392.7 (7600.7-11497.1)	268.6 (217.1-328.8)	275.9 (211.6-357)	6.5 (5-8.3)	4858.6 (3657-6280.8)	129.4 (97.2-168)	-2.82 (-3.15--2.48)	-3.3 (-3.72--2.87)
Lebanon	452 (351.3-571.4)	21 (16.5-26.5)	11966.4 (9258.1-15330.2)	497.1 (388.8-630.5)	798.3 (571.4-996.6)	15.4 (11-19.2)	18284.9 (13056.8-23177.8)	348.1 (249-443.6)	-0.67 (-0.9--0.45)	-0.83 (-1.11--0.56)
Lesotho	31.3 (22.4-42.1)	3.4 (2.5-4.5)	800.4 (552.4-1091)	78.5 (55.3-106.3)	65.8 (43-96.2)	5.5 (3.7-7.8)	1855.9 (1210-2757.3)	134 (88.4-196.5)	2.56 (2.13-2.98)	2.85 (2.37-3.34)
Liberia	29.2 (20.9-38.6)	2.8 (2.1-3.7)	752 (535.4-1003.7)	65.2 (46.9-86.5)	44.7 (30.2-62.3)	2.3 (1.6-3.1)	1272.6 (829.7-1818.9)	52.7 (35.3-74.2)	-0.67 (-0.81--0.54)	-0.75 (-0.89--0.61)

Libya	226.9 (170-303)	12.3 (9.2-16.2)	6340.5 (4711.7-8586)	300.8 (225.5-405)	593.1 (444.3-809.9)	11.7 (8.8-15.8)	16701 (12260.8-23329.5)	285.6 (213.2-395.4)	-0.14 (-0.29-0.02)	-0.19 (-0.34--0.03)
Lithuania	481 (381.4-584.7)	10.8 (8.6-13)	10171 (8093.1-12313.3)	229.2 (183.8-276.6)	352.9 (266.2-457.8)	5.8 (4.4-7.4)	6239.4 (4705.2-8194.7)	117.9 (88.6-155.2)	-2.26 (-2.48--2.03)	-2.43 (-2.75--2.11)
Luxembourg	29.3 (23.6-35.4)	5.5 (4.5-6.6)	681.9 (551.4-822.6)	132.7 (107.4-159.9)	13.5 (10.2-16.7)	1.3 (1-1.6)	276.3 (212.3-343.5)	29.2 (22.3-36.4)	-5.32 (-5.48--5.16)	-5.57 (-5.74--5.41)
Madagascar	205.2 (156-259.4)	4.2 (3.2-5.3)	5879.1 (4443.4-7660.4)	102.1 (77.9-130.9)	324.5 (205.7-474.9)	3 (1.9-4.3)	10197.2 (6426.9-15268.4)	75 (47.7-109.5)	-1.51 (-1.63--1.39)	-1.45 (-1.57--1.33)
Malawi	88.7 (64.8-117.5)	2.3 (1.7-2.9)	2737.3 (2000.7-3670)	60.2 (43.6-79.9)	133.9 (91.5-185.3)	1.8 (1.2-2.5)	4118.3 (2822.7-5822.4)	47.4 (32.5-66.2)	-1.08 (-1.27--0.9)	-1.15 (-1.38--0.92)

Malaysia	794.5 (635.4-971.6)	9.2 (7.3-11.2)	20322.6 (16215-25024.1)	210.1 (167.7-257.9)	1903 (1361.6-2502.5)	7.5 (5.4-9.8)	48016.6 (33863.7-63845.6)	170.2 (121.4-225.1)	-1.28 (-1.59--0.97)	-1.11 (-1.32--0.89)
Maldives	13.3 (10.3-17.2)	15.6 (12.2-19.6)	392.2 (302.5-515.6)	379.5 (295.4-487)	17.9 (14-22.7)	6.3 (4.8-8)	455.6 (353.5-578.1)	131.2 (101.4-167.5)	-3.82 (-4.07--3.57)	-4.38 (-4.66--4.1)
Mali	100.5 (71.5-133.2)	2.7 (2-3.6)	2685.3 (1893.3-3645.5)	61.4 (43.8-81.9)	210 (151.1-284)	2.7 (2-3.6)	5480.4 (3847.2-7510)	59.2 (42.5-80.5)	0.15 (0.04-0.26)	0 (-0.11-0.12)
Malta	30.2 (24.1-36.8)	7.2 (5.8-8.8)	709.9 (571.6-860.3)	166.7 (134.4-201.6)	20 (15.6-24.7)	2.2 (1.8-2.7)	407.2 (319.5-502.4)	52.4 (41.4-64.7)	-4.02 (-4.14--3.91)	-4.01 (-4.13--3.89)
Marshall Islands	2.5 (1.8-3.3)	15.3 (11.1-20.3)	77.4 (54.8-103.2)	404.5 (293-540.4)	5.6 (3.8-7.9)	15.6 (11-21.6)	186.9 (124-270.7)	426.1 (291-603.5)	0.08 (0.01-0.14)	0.18 (0.09-0.26)

Mauritania	45.2 (33.6-59.3)	4.8 (3.6-6.2)	1171.9 (857.7-1554.1)	111.3 (82.1-146.7)	51.2 (35.6-70.8)	2.6 (1.9-3.6)	1259.5 (843.1-1786.9)	57.6 (38.9-79.8)	-2.04 (-2.18--1.9)	-2.24 (-2.37--2.1)
Mauritius	103.9 (82.7-126.2)	14.8 (11.9-17.9)	2768.5 (2219.4-3371.7)	350.8 (281.1-425.3)	86.3 (64-112.5)	5.2 (3.9-6.7)	2008.5 (1469.1-2633.3)	115.7 (85.2-151)	-4.59 (-5.01--4.17)	-4.84 (-5.28--4.4)
Mexico	1574.6 (1201.9-1957.7)	4 (3.1-5)	38647.9 (29885.5-48088.8)	85.4 (65.8-105.7)	2491.9 (1730.9-3306.7)	2.2 (1.5-2.9)	60176.8 (41566.2-80366.5)	49.6 (34.2-66.3)	-2.49 (-2.83--2.15)	-2.27 (-2.59--1.96)
Micronesia (Federated States of)	7.4 (5-10.4)	15.8 (10.8-22.1)	235.6 (160.1-343.4)	436.9 (300-622.1)	11.5 (7.3-16.9)	16 (10.6-22.6)	366.4 (223.6-549.7)	433.5 (273.9-639.4)	0.07 (0.02-0.12)	0.01 (-0.04-0.06)
Monaco	2.9 (2.1-3.9)	4.3 (3.1-5.6)	58.9 (42.3-78.1)	101.8 (74.1-134.2)	1.6 (1.2-2.1)	1.7 (1.3-2.2)	30.8 (22.6-40.6)	41.1 (29.6-54.8)	-3.25 (-3.49--3)	-3.2 (-3.45--2.95)

Mongolia	181.4 (140.4-230.8)	18.9 (14.6-23.9)	4675.4 (3537.1-5954.4)	426.9 (327.6-544.6)	237.1 (176.2-314.1)	12 (9.1-15.4)	6706.7 (4864.2-9123.8)	257.3 (191.4-339)	-2.51 (-2.99--2.02)	-2.72 (-3.19--2.25)
Montenegro	46.3 (37.1-56.8)	7.8 (6.2-9.5)	1077 (859.2-1310.1)	170.1 (136.2-206.1)	90.5 (68.9-114)	9.5 (7.3-11.9)	1846.3 (1376.1-2357.1)	192.6 (145.2-245.3)	1.05 (0.73-1.38)	0.63 (0.24-1.02)
Morocco	2279.7 (1767.9-2798.9)	17.6 (13.8-21.6)	61223.5 (47445.1-75894.1)	414.9 (323.1-510.9)	3625.9 (2655.2-4635.3)	12.4 (9.2-15.8)	91505.9 (65679.7-121315.9)	277.4 (201.3-359)	-1.47 (-1.64--1.3)	-1.64 (-1.76--1.52)
Mozambique	92.1 (64.9-122.9)	1.6 (1.1-2.1)	2663 (1860-3625.3)	39.4 (27.9-53)	222.1 (148.1-314.8)	2.1 (1.4-2.9)	6593.2 (4283.9-9538.6)	51.6 (34.3-73.5)	1.49 (1.24-1.75)	1.6 (1.3-1.89)
Myanmar	1971.1 (1407.2-2651.6)	8.7 (6.4-11.5)	59549.7 (41250.5-82191.7)	225.9 (160.8-307.9)	1870.3 (1411.9-2407.8)	4.4 (3.3-5.6)	47497.8 (35013.9-62363.3)	97.9 (73.4-127.7)	-2.69 (-2.81--2.56)	-3.23 (-3.37--3.09)

Namibia	31.2 (22.1-40.9)	4.6 (3.4-6)	792.9 (552.5-1063.3)	106.8 (75.6-143.3)	43.9 (30.3-61)	3.3 (2.3-4.6)	1102.6 (754.4-1582.9)	74.4 (50.5-104.7)	-1.49 (-1.74--1.24)	-1.64 (-1.96--1.32)
Nauru	0.9 (0.6-1.2)	21.4 (15.9-28.5)	31.9 (22.4-44.4)	591.7 (428.7-803.6)	1 (0.7-1.4)	21.6 (16.1-28.7)	37.9 (26.8-52.7)	598 (435.9-809.8)	-0.05 (-0.4-0.3)	-0.04 (-0.43-0.35)
Nepal	476.7 (342.2-652.9)	5.1 (3.7-6.9)	14714.3 (10360.9-20423.5)	133.2 (95-182.9)	1018.4 (722.8-1372)	4.9 (3.5-6.5)	26669.1 (18861.1-36315)	113.4 (80.2-152.6)	-0.2 (-0.35--0.04)	-0.57 (-0.75--0.4)
Netherlands	1094 (878.7-1331.5)	5.6 (4.5-6.8)	26237.7 (21116.5-31825.7)	140.9 (113.3-170.8)	358.5 (281.7-444.6)	1.1 (0.9-1.3)	7506 (5918.3-9257.7)	25.7 (20.5-31.4)	-6.4 (-6.76--6.04)	-6.6 (-6.96--6.24)
New Zealand	216.8 (173-262.5)	5.7 (4.6-6.9)	5536 (4431.5-6682.7)	149.2 (118.2-179.6)	119.4 (92.8-147.7)	1.6 (1.2-1.9)	2631 (2050.4-3242.6)	39 (30.5-48.6)	-4.93 (-5.23--4.63)	-5.11 (-5.42--4.79)

Nicaragua	51.4 (40.5-63.8)	3.5 (2.8-4.4)	1330.5 (1042.6-1657)	80 (63-99)	163.1 (120.1-214)	4.2 (3.1-5.4)	3688.5 (2719.7-4900.4)	81.9 (60.3-107.8)	0.39 (0.18-0.6)	-0.07 (-0.23-0.08)
Niger	73.2 (51.1-99.6)	2.8 (2-3.8)	2093.6 (1436.5-2920.4)	66.7 (46.5-91.4)	192.3 (134.4-264.1)	2.7 (1.9-3.6)	5391.8 (3677.7-7577.2)	62.1 (43.5-86.1)	-0.19 (-0.4-0.03)	-0.27 (-0.49--0.05)
Nigeria	1091.2 (704.9-1686)	2.7 (1.7-4)	28350.4 (17841.5-44877.4)	60.7 (39-94.7)	1694 (1144.1-2385.3)	2.1 (1.5-3)	45245.7 (30055.6-66498.4)	47.3 (31.9-67.1)	-0.99 (-1.16--0.83)	-1.14 (-1.34--0.95)
Niue	0.3 (0.3-0.4)	15.5 (11.8-20.2)	8.3 (6.2-10.9)	396.5 (297-524)	0.3 (0.2-0.4)	13.9 (10.2-17.9)	7.5 (5.3-9.9)	350.2 (248.2-472.1)	-0.52 (-0.63--0.4)	-0.62 (-0.75--0.49)
North Macedonia	230.8 (188.4-277.4)	13.3 (10.8-16)	5730.8 (4626.1-6873.9)	298.1 (243.4-356.6)	312.2 (227.8-408.5)	11.2 (8.4-14.5)	6680.4 (4880.6-8843)	218 (161-286.6)	-0.94 (-1.22--0.66)	-1.59 (-1.84--1.33)

Northern Mariana Islands	1.3 (1-1.8)	7 (5.3-9.1)	47.3 (33.2-65.8)	176.9 (131.5-235.2)	4.2 (3.1-5.5)	8 (6.2-10.1)	121.1 (88.9-159.3)	201.3 (151.3-260.7)	0.82 (0.7-0.94)	0.78 (0.67-0.89)
Norway	456.4 (365.7-558.2)	7.1 (5.7-8.6)	10123.9 (8128.4-12329.2)	174.2 (140.7-212.3)	112.4 (89.8-138.8)	1.2 (0.9-1.4)	2256.8 (1798.1-2783.4)	26.5 (21-32.6)	-6.74 (-7.01--6.48)	-6.94 (-7.2--6.68)
Oman	117.3 (86.2-157.5)	19.6 (14.5-25.8)	3310.1 (2382.2-4497.2)	441.6 (325.6-589.3)	163 (126.4-203.5)	12.3 (9.6-15.3)	4468.1 (3411.5-5629.4)	235.6 (182.6-294.8)	-1.49 (-1.6--1.38)	-2.09 (-2.31--1.88)
Pakistan	4541.8 (3465.8-5675.8)	8 (6-10)	130304.2 (100036.5-162442.6)	207.2 (158.4-258.7)	10302.4 (7704.3-13444)	9.1 (6.9-11.9)	326715.3 (238388.1-428463.5)	241 (179.5-313.5)	0.15 (-0.16-0.46)	0.18 (-0.15-0.51)
Palau	1.6 (1.2-2.3)	16.8 (12.3-22.9)	49.6 (34.8-69.9)	447.1 (321.1-620.6)	3.2 (2.4-4.4)	15.1 (11.3-20.1)	98.4 (71.5-133.5)	406.3 (299-545.6)	-0.44 (-0.49--0.39)	-0.37 (-0.42--0.32)

Palestine	153.1 (111.6-201.1)	18.3 (13.5-24)	3920 (2855.1-5177.9)	431.2 (312.3-568.9)	255.3 (198.1-330.2)	11.6 (9-14.6)	6856.4 (5290.1-8721.7)	261.1 (202.2-337.5)	-2.02 (-2.19--1.85)	-2.17 (-2.34--1.99)
Panama	45.6 (36.3-55.3)	3.1 (2.5-3.8)	1075 (864.8-1303.2)	69.1 (55.7-83.7)	50.2 (35.4-67.9)	1.2 (0.8-1.6)	1149.8 (799-1610.1)	27.5 (19.1-38.7)	-3.2 (-3.49--2.9)	-2.95 (-3.25--2.64)
Papua New Guinea	157 (102-244.5)	8.4 (5.6-12.9)	4966 (3202.9-7768.1)	223 (144.8-345)	537.2 (351.3-795.8)	10.9 (7.3-15.9)	17383.6 (11296.3-26157.2)	288.8 (190.2-426)	1.12 (0.92-1.32)	1.14 (0.93-1.35)
Paraguay	97.9 (77.1-120.4)	4.5 (3.5-5.5)	2525.4 (2002.3-3109.4)	106.5 (84.6-131.1)	191.5 (134.7-259.4)	3.5 (2.5-4.7)	4879.8 (3402.5-6754.8)	83.4 (58.1-116.3)	-0.78 (-1.12--0.44)	-0.77 (-1.11--0.42)
Peru	298.3 (225.2-385.6)	2.5 (1.9-3.3)	7948.3 (5913.2-10221.5)	60.3 (45-77.4)	273 (177.9-381.3)	0.8 (0.5-1.2)	6504.5 (4182.9-9233.2)	19.6 (12.6-28)	-4.21 (-4.63--3.79)	-4.17 (-4.6--3.73)

Philippines	1368.8 (1065.1-1716.4)	5.9 (4.7-7.4)	33543.3 (26314.2-44180.5)	111.1 (87-141.7)	6118.1 (4585.2-7943.6)	8.3 (6.3-10.7)	170467.2 (124956.5-221639.3)	197.9 (146.4-256.3)	1.8 (1.3-2.31)	2.69 (2.04-3.34)
Poland	6350.7 (5098.4-7651.7)	15.2 (12.4-18.2)	145093.5 (117746-173429.7)	338.2 (279.1-405.5)	3071 (2347.4-3908.4)	4.3 (3.3-5.4)	55573.4 (42095.2-71948.2)	84.3 (64-110.8)	-4.83 (-5.03--4.63)	-5.25 (-5.47--5.02)
Portugal	651.7 (532.7-780.7)	5 (4.1-5.9)	14578.2 (12023.9-17330.4)	111.6 (92.6-131.9)	319 (248.7-393.8)	1.3 (1.1-1.6)	6069.8 (4894.6-7392.1)	30.6 (24.7-37.1)	-5.32 (-5.63--5.01)	-5.25 (-5.58--4.93)
Puerto Rico	144.1 (116.6-173.3)	4.1 (3.3-4.9)	3578.6 (2872.9-4299.6)	100.3 (80.4-120.4)	99.8 (70.3-134.5)	1.5 (1-2)	2202.3 (1542.3-2987.6)	38.1 (26.6-52.1)	-3.92 (-4.16--3.69)	-3.76 (-3.99--3.54)
Qatar	20.5 (15.8-26.8)	22.3 (16.8-28.7)	636.5 (492.3-836.4)	464.1 (354.1-607)	54.4 (38.7-74.8)	10.3 (7.5-13.5)	1673.8 (1187.6-2324.6)	172.3 (124.4-229.4)	-3.07 (-3.34--2.8)	-3.96 (-4.24--3.68)

Republic of Korea	1594.6 (1283.1-1905.2)	6.1 (4.9-7.3)	45053.9 (36131.4-53717.4)	134.1 (108-159.7)	679.2 (527.3-850.7)	0.8 (0.6-1)	14087.1 (11189.8-17649.2)	16.4 (13-20.4)	-6.95 (-7.38--6.52)	-7.11 (-7.59--6.63)
Republic of Moldova	528.1 (433.5-637.9)	13.7 (11.3-16.4)	11623.1 (9535.8-13929.7)	269.3 (220.6-321.3)	418.5 (326.4-519.6)	7.3 (5.7-9)	8717.4 (6773.9-10902.1)	154 (120-192.3)	-2.64 (-2.98--2.29)	-2.26 (-2.64--1.87)
Romania	3336.5 (2674.6-4046.7)	13.4 (10.8-16.2)	71933.3 (58399.5-86867.2)	265.9 (217.5-320.9)	2768.3 (2105.1-3609.8)	7.3 (5.5-9.4)	51011.5 (38435.6-66587.5)	147.5 (112.1-192)	-2.75 (-3.06--2.44)	-2.79 (-3.14--2.43)
Russian Federation	18400.7 (14690.6-22487.2)	10.9 (8.8-13.2)	426589.8 (341541.9-515393)	239.1 (193.1-288.6)	19114.8 (14996.1-23799.6)	8.2 (6.4-10.3)	424244.6 (328541.7-531945.9)	188 (145.3-236.7)	-1.54 (-2.23--0.85)	-1.5 (-2.3--0.69)
Rwanda	70 (49.3-97.2)	2.4 (1.7-3.3)	2117.9 (1477.2-2951.5)	64 (44.8-88.6)	89.9 (56.5-131.3)	1.6 (1-2.2)	2609.7 (1623.1-3963.8)	37.5 (23.6-55.5)	-2.25 (-2.57--1.93)	-2.74 (-3.11--2.37)

Saint Kitts and Nevis	3 (2.4-3.8)	8.6 (6.8-10.4)	70 (54.4-86.5)	205.4 (162.7-250.7)	1.8 (1.3-2.3)	2.8 (2.1-3.6)	45.5 (31.3-60.8)	64 (45.2-84.5)	-4.27 (-4.62--3.91)	-4.76 (-5.17--4.35)
Saint Lucia	3.2 (2.5-3.9)	3.9 (3.1-4.7)	78.7 (63-96.5)	90.7 (72.3-110.9)	2.9 (2.2-3.7)	1.4 (1-1.8)	73.5 (53.6-95.4)	33.4 (24.6-43)	-4.05 (-4.51--3.59)	-3.86 (-4.28--3.44)
Saint Vincent and the Grenadines	3.3 (2.6-4.2)	4.8 (3.8-6)	79.4 (62.3-97.7)	111 (87.4-137.3)	4 (3.1-5.1)	3.1 (2.4-4)	94 (71.2-118.8)	69.6 (52.8-87.7)	-1.58 (-1.87--1.29)	-1.78 (-2.04--1.51)
Samoa	14 (10.6-18.2)	16.5 (12.9-21.1)	386.4 (286.3-519.3)	414.8 (311.2-551.5)	22.1 (16.3-28.7)	15.3 (11.4-19.6)	621.5 (435.4-826.3)	390.6 (279.2-511.7)	-0.27 (-0.31--0.24)	-0.2 (-0.25--0.15)
San Marino	0.8 (0.6-1)	2.4 (1.8-3)	16.3 (12.5-20.9)	52.3 (40-67)	0.8 (0.5-1.2)	1.2 (0.7-1.8)	15.4 (9.3-23.7)	28.3 (16.7-44.2)	-2.44 (-2.67--2.21)	-2.21 (-2.46--1.96)

Sao Tome and Principe	0.9 (0.6-1.1)	1.4 (1.1-1.8)	23.4 (16.8-31.2)	35.3 (25.6-46.7)	1.6 (1.1-2.2)	1.6 (1.1-2)	47.7 (32.1-66.3)	38.4 (26.3-51.5)	0.03 (-0.19-0.25)	-0.05 (-0.35-0.26)
Saudi Arabia	783.9 (575-1043.6)	13.8 (10.2-18)	22578.3 (16227.1-30879.1)	329.2 (240.3-441.2)	2175.1 (1637.4-2826.6)	11.5 (8.8-14.5)	71570.6 (52772-95231.4)	282.4 (214.2-363)	-0.54 (-0.77--0.3)	-0.34 (-0.6--0.08)
Senegal	206.9 (154.7-266.7)	7.2 (5.4-9.2)	5072.1 (3754.8-6494.5)	152.6 (113.7-194.2)	346.8 (250.5-466.8)	5.1 (3.8-6.7)	8511.1 (5995.5-11737.6)	108.6 (77.5-148.8)	-1.29 (-1.41--1.18)	-1.26 (-1.41--1.12)
Serbia	1289.9 (1042-1561.8)	12.6 (10.2-15.3)	28847.3 (23366.9-34797.4)	258.2 (210.4-311.7)	1325.1 (964.8-1737.6)	8.9 (6.6-11.5)	24287.7 (17577.9-31701.7)	162.2 (119-211.2)	-1.73 (-2.1--1.35)	-2.25 (-2.66--1.83)
Seychelles	4.1 (3.2-5)	7.2 (5.7-8.9)	96.5 (75.9-119.2)	171.4 (135-212.4)	5.1 (4-6.4)	4.9 (3.8-6.1)	127.4 (99-162.3)	110 (86.1-137.7)	-1.66 (-1.8--1.52)	-1.89 (-2.04--1.74)

Sierra Leone	102.8 (70.3-144.9)	5.8 (4-8.2)	2406.2 (1605-3445.9)	123.1 (82.6-175)	166.2 (111-236.6)	5.1 (3.5-7.1)	4247.1 (2801-6248.9)	110.2 (73.4-160.2)	-0.21 (-0.37--0.06)	-0.15 (-0.33-0.03)
Singapore	89 (70.1-109)	4 (3.2-4.9)	2494.8 (1950.9-3060.9)	100.7 (78.7-123.9)	83.9 (66.4-104.6)	1.1 (0.8-1.3)	2153 (1688.6-2696.3)	26.3 (20.7-32.8)	-4.81 (-4.95--4.68)	-4.83 (-4.94--4.71)
Slovakia	1040.1 (832.1-1266.4)	18 (14.5-21.7)	22739.2 (18511.6-27734.4)	387.3 (316.4-468.6)	672.1 (481.5-878.3)	7.5 (5.4-9.7)	12401.9 (8737.3-16587.4)	139.9 (99.4-187.2)	-2.99 (-3.16--2.82)	-3.53 (-3.72--3.34)
Slovenia	156.3 (112.2-210.9)	6.6 (4.7-8.8)	3622.7 (2579.6-4946.8)	150.1 (107.1-204.6)	96.3 (70.4-129.5)	2.2 (1.6-2.9)	1834.2 (1338.3-2482.1)	47 (34.3-64.6)	-4.22 (-4.37--4.07)	-4.4 (-4.55--4.25)
Solomon Islands	42.2 (29.8-58.6)	28.1 (20.4-37.5)	1446.7 (1009.6-2026.1)	829.7 (582.7-1138.3)	98.2 (71.2-131.3)	28.3 (21-36.6)	3440.8 (2453.1-4694.7)	827.1 (600.9-1102.4)	-0.02 (-0.07-0.04)	-0.02 (-0.07-0.04)

Somalia	78.9 (52.8-113.6)	3.2 (2.2-4.5)	2486.3 (1611.5-3707.7)	79.8 (53.3-114.7)	202.3 (133.2-292.5)	3.1 (2.1-4.5)	6387.7 (4203.1-9288.2)	78.6 (51.5-113.9)	0.22 (0.11-0.32)	0.16 (0.08-0.25)
South Africa	976.7 (771.3-1178.7)	4.8 (3.7-5.8)	27545.1 (21927.8-33812.7)	117.8 (93.3-143)	1213.2 (938.2-1508.1)	2.9 (2.3-3.6)	30167.2 (23211.8-37508.4)	64 (49.4-79.4)	-1.82 (-2.22--1.43)	-2.21 (-2.62--1.8)
South Sudan	47.5 (33.1-63.7)	2.2 (1.6-2.9)	1233.4 (826-1695)	48.8 (33.3-66.5)	71.4 (46.1-105.3)	2.1 (1.3-3)	1925.1 (1206.3-2911.5)	45.5 (29.2-67)	0.02 (-0.1-0.13)	-0.09 (-0.2-0.02)
Spain	2111.5 (1691.1-2545.6)	4.1 (3.3-4.8)	48416 (39227.8-58037.2)	97.3 (78.9-115.9)	1250 (985.6-1536.4)	1.3 (1-1.6)	25904.3 (20613.8-31696.3)	32.5 (26.1-39.8)	-4.14 (-4.29--3.99)	-4.03 (-4.18--3.87)
Sri Lanka	669 (514.4-833.2)	6.6 (5.1-8.2)	18270.8 (14212.6-22717.4)	153.1 (119.3-189.9)	873.6 (574.2-1220.7)	3.7 (2.4-5.1)	20194.1 (13219.5-28620.2)	79.3 (52.6-111.5)	-1.92 (-2.09--1.76)	-2.38 (-2.59--2.16)

Sudan	1849.2 (1360.2-2380.4)	20.3 (15.1-25.9)	53240 (38790.2-68687.5)	513.9 (377.3-662)	2316.8 (1654.3-3124.6)	12.7 (9.2-16.9)	65477.3 (44131.2-92512.9)	303.8 (214.1-415.5)	-1.8 (-1.93--1.67)	-1.99 (-2.12--1.86)
Suriname	22.4 (18.1-27.2)	8.7 (7-10.6)	584.4 (465.2-712.1)	211.6 (170.1-258)	28.6 (21.3-37.1)	4.8 (3.6-6.2)	747.9 (553.3-975.7)	118.7 (87.9-154.7)	-2.39 (-2.77--2.01)	-2.39 (-2.76--2.02)
Sweden	684.1 (536.3-840.6)	4.7 (3.8-5.7)	14476.5 (11558.4-17680.3)	110.6 (88.9-135.8)	264.6 (213.8-324.2)	1.3 (1-1.5)	5082.4 (4061.1-6165.7)	28.9 (23.2-35.1)	-4.77 (-4.98--4.57)	-4.87 (-5.08--4.67)
Switzerland	487.3 (389.6-589.8)	4.7 (3.8-5.7)	10578 (8591.4-12798.6)	111.2 (90.4-134.7)	216.1 (165.9-268.2)	1.2 (0.9-1.4)	3922.8 (3111.5-4753.1)	25.3 (20-30.6)	-5.48 (-5.75--5.22)	-5.73 (-5.97--5.48)
Syrian Arab Republic	1140 (856.8-1488.4)	21.7 (16.4-28.1)	33594 (24973.4-44783.1)	548.7 (409.7-723.9)	1963.8 (1404.1-2691)	17.3 (12.7-23.3)	52666.2 (37100.3-73223.5)	399.8 (286.7-548.3)	-1.26 (-1.46--1.06)	-1.55 (-1.78--1.31)

Taiwan (Province of China)	666.7 (544-800)	5 (4.1-6)	15467.3 (12618.9-18587.5)	97.7 (80.8-115.9)	710.1 (525.7-931.9)	1.8 (1.3-2.4)	14604.8 (10725.2-19246.6)	38.2 (28.1-50.2)	-3.7 (-3.93--3.46)	-3.27 (-3.49--3.06)
Tajikistan	313.2 (253-376.7)	11.4 (9.1-13.9)	7704.9 (6165.6-9246.8)	266.5 (212.6-319.7)	586.3 (447.4-757.6)	15.1 (11.5-19.2)	15292.6 (11496.1-19961.6)	301.6 (231.8-388.5)	1.18 (1.01-1.35)	0.44 (0.25-0.63)
Thailand	1394.8 (1093.6-1751.2)	4.5 (3.5-5.7)	36242 (28506.7-45708.6)	94.2 (74.6-118.5)	1923.8 (1299.1-2631.9)	1.9 (1.3-2.6)	43959.6 (29813.8-61827.5)	44.3 (30.2-61.7)	-3.54 (-3.76--3.32)	-3.32 (-3.58--3.07)
Timor-Leste	14 (10.1-18.9)	5.7 (4.2-7.6)	402.9 (283.8-558.2)	125.5 (90.4-169.4)	52 (36.1-71)	7.1 (5.1-9.7)	1256.1 (824.3-1733.1)	151.9 (103.5-207.9)	0.96 (0.82-1.09)	0.74 (0.58-0.9)
Togo	46.7 (34.8-60.4)	3.9 (3-5.1)	1365.2 (1002.8-1799)	94.5 (70.1-122.8)	116.7 (82.1-162.8)	3.2 (2.3-4.3)	3575.9 (2466.7-5137.4)	80.4 (56.6-112.8)	-0.72 (-0.76--0.68)	-0.56 (-0.61--0.5)

Tokelau	0.2 (0.1-0.2)	13.5 (9.9-18)	4.4 (3.2-6.1)	345.1 (248.6-474.8)	0.2 (0.1-0.2)	12.3 (8.8-16.2)	4.2 (2.9-5.7)	312.5 (217.2-427.4)	-0.31 (-0.44--0.17)	-0.34 (-0.47--0.2)
Tonga	4.3 (3.3-5.6)	8.1 (6.2-10.5)	119.3 (89.7-156)	201.4 (151.9-262.8)	6 (4.3-7.9)	7.6 (5.5-9.9)	152.5 (109.7-205.7)	187 (134.5-251.4)	-0.27 (-0.41--0.13)	-0.28 (-0.39--0.16)
Trinidad and Tobago	67.4 (53.9-82.6)	8.3 (6.7-10.1)	1770.7 (1408.7-2161.6)	203.6 (162.4-248.9)	79.1 (54.5-109)	4.3 (3-5.9)	1914.9 (1295.2-2695.8)	103.4 (70.2-145)	-2.83 (-3.06--2.59)	-2.94 (-3.18--2.69)
Tunisia	725.6 (562.7-903.2)	16 (12.5-19.8)	17996.1 (14020.4-22658)	344.4 (268.4-427.3)	1248.4 (849.4-1725.3)	10.5 (7.2-14.4)	28209.1 (19072.7-39131.7)	220.4 (150-303.8)	-1.73 (-1.86--1.6)	-1.82 (-1.95--1.7)
Turkey	5637.5 (4483.2-6908.1)	16.5 (13.1-20.1)	144042.2 (114283-179305.4)	378.8 (301.9-466.8)	4859.3 (3570.9-6457.6)	5.7 (4.2-7.6)	104879.8 (76102-140508)	117.3 (85.5-157.1)	-4.05 (-4.35--3.74)	-4.63 (-4.96--4.3)

Turkmenistan	438.3 (356.9-517.7)	25.7 (21-30.6)	10620.4 (8612.6-12529.1)	544.5 (443.8-641.9)	792.9 (603-1018.3)	22.9 (17.7-29.3)	19320.9 (14577.7-25192.9)	482.8 (368.8-625.8)	-1.33 (-1.77--0.89)	-1.39 (-1.87--0.91)
Tuvalu	1.2 (0.8-1.6)	17.7 (12.9-23.5)	34.9 (24.7-48.1)	470.7 (338-643.4)	1.6 (1.1-2.2)	15.9 (11.6-21.6)	45.5 (32.4-63)	423.6 (304.5-578.4)	-0.35 (-0.41--0.29)	-0.31 (-0.37--0.26)
Uganda	87.2 (58.2-121)	1.5 (1-2)	2357.6 (1553-3324)	33.6 (22.2-46.8)	204.9 (129.5-289.2)	1.5 (0.9-2.1)	6050.1 (3783-8779.9)	36.1 (22.8-51.2)	-0.45 (-0.73--0.17)	-0.37 (-0.72--0.01)
Ukraine	8853.3 (7068-10855.9)	13.1 (10.5-15.8)	182174.9 (147459.9-220476.1)	259.7 (210.6-312)	11229.4 (8695.7-14106.4)	14.7 (11.4-18.4)	231985.6 (178536.7-293482.5)	319.2 (246.1-405.2)	-0.07 (-0.48-0.36)	0.13 (-0.36-0.62)
United Arab Emirates	63.1 (46.2-84.5)	17.3 (13-22.5)	2095.6 (1502.3-2873.6)	371.2 (275.7-489.2)	416.4 (281.2-614.4)	10.9 (7.8-14.6)	15020.6 (10005.7-22378.9)	241.8 (168.3-331.4)	-1.84 (-2.31--1.37)	-1.75 (-2.1--1.39)

United Kingdom	6617.3 (5324-8055.1)	7.7 (6.2-9.2)	152258.5 (121542.2-184026.2)	190 (152.9-227.9)	2125.4 (1689.8-2591.3)	1.8 (1.4-2.2)	46532.3 (37120.2-56525.5)	44.4 (35.4-54.1)	-5.69 (-6.02--5.35)	-5.58 (-5.92--5.24)
United Republic of Tanzania	208.2 (148.6-274.8)	2.2 (1.6-2.8)	5512 (3884.7-7261.9)	47.5 (34.1-61.8)	547.3 (367.1-762.8)	2.4 (1.6-3.3)	14618.7 (9592.3-20912.2)	53.9 (35.8-76.3)	0.28 (0.21-0.35)	0.36 (0.29-0.44)
United States of America	19081.8 (15708.8-22676.4)	6.2 (5.2-7.4)	472385.8 (392506.5-559175.3)	163.5 (135.4-193.7)	11473 (9300.8-13741.3)	2.1 (1.7-2.6)	272867.6 (221878.6-330789.3)	56.5 (46-68.1)	-4.28 (-4.59--3.98)	-4.19 (-4.48--3.89)
United States Virgin Islands	4.9 (3.8-6.2)	6 (4.7-7.6)	133.4 (100.7-172)	143 (109.9-183.4)	8.6 (6.7-10.9)	4.9 (3.8-6.1)	197.8 (149-252.6)	113.5 (85.6-147.2)	-0.56 (-0.75--0.36)	-0.65 (-0.87--0.43)
Uruguay	322.1 (254.7-393.2)	8.4 (6.7-10.1)	6629.1 (5273.2-8135.1)	175 (139.9-212.7)	172.9 (137.2-210.9)	3.1 (2.4-3.7)	3319.8 (2654.2-4031.8)	66.3 (53.1-80.2)	-3.69 (-3.84--3.53)	-3.59 (-3.74--3.44)

Uzbekistan	1260 (998.7-1534.1)	11.9 (9.5-14.6)	28731.3 (22793.8-34446.6)	249.5 (199.7-301.8)	3390.7 (2549.4-4351.1)	24.2 (18.8-30.3)	89008.4 (67489-114334.9)	451.9 (348.3-570.8)	2.61 (1.92-3.31)	2.09 (1.42-2.76)
Vanuatu	8.1 (5.6-11.8)	12 (8.4-16.8)	258.2 (171.6-376.6)	329.3 (222.2-477.8)	23.7 (16.5-33.2)	13.3 (9.4-18.5)	750 (508.7-1075.6)	375.1 (256-534.2)	0.04 (-0.13-0.21)	0.09 (-0.09-0.26)
Venezuela (Bolivarian Republic of)	724.3 (566.4-882.6)	7.7 (6-9.5)	19019 (14956-23111.3)	180.2 (141.6-219.4)	1424.3 (973.8-1964.5)	5 (3.4-6.9)	34572.4 (23159.2-47643)	115.2 (77.6-158.5)	-1.87 (-2.05--1.68)	-1.89 (-2.07--1.71)
Viet Nam	2231.3 (1687.5-2907.4)	5.9 (4.5-7.7)	52949.5 (39600.8-70456.5)	129.3 (97.7-170.5)	3974.9 (2930-5142.3)	4.7 (3.5-6.1)	90716.2 (66098.2-120915.4)	95.6 (69.8-126)	-0.98 (-1.1--0.85)	-1.19 (-1.34--1.05)
Yemen	1011.3 (723.1-1361.4)	21.5 (15.8-28.3)	29479.8 (20712.9-40248)	527.9 (376.9-713.1)	2250 (1656.8-3029.8)	17.8 (13.4-23.7)	63791.6 (45717-88079)	418.9 (306.4-565.5)	-0.85 (-0.96--0.75)	-1.03 (-1.15--0.92)

Zambia	85.3 (59.8-116.8)	3 (2.2-4)	2604.3 (1815.3-3613.4)	76.9 (54-105.9)	156.5 (104.9-221.2)	2.4 (1.6-3.3)	4836.5 (3213-7018.4)	58.4 (39.1-83.1)	-1.22 (-1.47--0.98)	-1.43 (-1.72--1.14)
Zimbabwe	150.8 (116-189.4)	4.1 (3.2-5.1)	3875.3 (2958.4-4925.4)	91.5 (70.3-114.8)	338.7 (239-458.1)	5.3 (3.8-7.1)	9246.9 (6518.8-12911.9)	123.4 (87-168)	1.64 (1.3-1.98)	1.95 (1.57-2.33)

ASDR: age-standardized disability-adjusted life year rate; ASMR: age-standardized mortality rate; CI: confidence interval; DALYs: disability-adjusted life-years; EAPC: estimated annual percentage change; UI: uncertainty interval.

Supplementary Table 2. Ischemic heart disease burden attributable to secondhand smoke in different age groups in 2019.

**Age-specific**	**Death cases, n (95% UI)**	**ASMR per 105, n (95% UI)**	**DALYs, n (95% UI)**	**ASDR per 105, n (95% UI)**
25-29 years	3072.2 (1964.7-4398.4)	0.5 (0.3-0.7)	195539.4 (124889.5-280407.4)	32.8 (21-47.1)
30-34 years	5212.7 (3628.1-6946.2)	0.9 (0.6-1.2)	305157.3 (212354.4-406107.1)	50.9 (35.4-67.8)
35-39 years	7151.4 (5174.5-9416.5)	1.3 (1-1.8)	382825.9 (276420.8-504203.5)	71.3 (51.5-93.9)
40-44 years	11616.8 (8470.4-15225.8)	2.4 (1.7-3.1)	563826.7 (412158.3-738049.1)	115.2 (84.2-150.9)
45-49 years	17681.6 (13285.5-23286.7)	3.8 (2.8-4.9)	773267 (580183.6-1016423.6)	164.1 (123.1-215.7)
50-54 years	25508.2 (18909-32271.5)	5.9 (4.4-7.4)	992646.3 (734203.2-1259617.2)	228.4 (168.9-289.8)
55-59 years	33376.5 (25607.4-41955.8)	9 (6.9-11.3)	1140834.4 (876107.1-1435022.9)	308.2 (236.7-387.6)

60-64 years	37593.8 (28361.1-47285.3)	12 (9.1-15.1)	1108442.1 (835504.2-1394792.8)	354.9 (267.5-446.6)
65-69 years	40736.3 (30176-51601.4)	15.7 (11.6-19.9)	1019775.4 (759871.9-1285253.2)	393.7 (293.3-496.2)
70-74 years	41277.3 (29808.1-53849.2)	22 (15.9-28.7)	852089.7 (612856.8-1109545.6)	454.3 (326.7-591.5)
75-79 years	45397.9 (32780.8-59599.7)	35.6 (25.7-46.7)	740674.7 (534870.8-970667.6)	580.1 (418.9-760.3)
80-84	54382.1 (36972.7-73011.3)	64.6 (43.9-86.7)	691947.1 (471973.4-927188.6)	821.8 (560.6-1101.2)
85-89	47087.6 (30870.3-66386.8)	108.8 (71.3-153.4)	473860.3 (310691.5-667772.7)	1094.9 (717.9-1543)
90-94	25919.1 (16928.8-36021.4)	154.1 (100.6-214.2)	226544.1 (148130-314447.7)	1346.9 (880.7-1869.5)
95+ years	9133.8 (5458.1-13096.2)	184.4 (110.2-264.5)	74844.1 (44879.8-107177.4)	1511.4 (906.3-2164.3)

ASDR: age-standardized disability-adjusted life year rate; ASMR: age-standardized mortality rate; DALYs: disability-adjusted life-years; UI: uncertainty interval.

©2024 Yang X. et al.
